# Supplementary material for: Key activity descriptors of nickel-iron oxygen evolution electrocatalysts in the presence of alkali metal cations
Source: Nat Commun. 2020 Dec 2;11:6181. doi: 10.1038/s41467-020-19729-2 (PMC7710789; doi:10.1038/s41467-020-19729-2)
Supplement: Supplementary file 1 — Supplementary Information [file 41467_2020_19729_MOESM1_ESM.pdf]

Supplementary Information

Key activity descriptors of nickel-iron oxygen evolution electrocatalysts in the presence of alkali metal cations

Mikaela Görlin,[1,2*] Joakim Halldin Stenlid,[1] Sergey Koroidov,[1] Hsin-Yi Wang,[1] Mia Börner,[1] Mikhail Shipilin,[1] Aleksandr Kalinko,[3,4] Vadim Murzin,[4,5] Olga V. Safonova,[6] Maarten Nachtegaal,[6] Abdusalam Uheida,[7] Joydeep Dutta,[7] Matthias Bauer, [3] Anders Nilsson, [1] and Oscar Diaz-Morales [1,8*]

[1] Department of Physics, AlbaNova University Center, Stockholm University, SE-106 91 Stockholm, Sweden

[2] Department of Chemistry - Ångström laboratory, Uppsala University, Box 538, SE-751 21 Uppsala, Sweden

[3] Department of Chemistry and Center for Sustainable Systems Design (CSSD), University of Paderborn, Warburger Strasse 100, D-33098 Paderborn, Germany

[4] Deutsches Elektronen-Synchrotron DESY, Notkestraße 85, D-22607 Hamburg, Germany

[5] Bergische Universität Wuppertal, Gaußstraße 20, D-42119, Wuppertal, Germany

[6] Paul Scherrer Institute, CH-5232 Villigen, Switzerland

[7] Functional Materials, Department of Applied Physics, School of Engineering Sciences, KTH Royal Institute of Technology, Hannes Alfvéns väg 12, SE-114 19 Stockholm, Sweden

[8] Applied Electrochemistry, School of Chemical Science and Engineering, KTH Royal Institute of Technology, SE-100 44 Stockholm, Sweden

*Corresponding authors: mikaela.gorlin@kemi.uu.se, oadm@kth.se

Supplementary Note 1. Electrolyte purification. The alkali hydroxides (0.1 M LiOH, NaOH, KOH, RbOH, CsOH) were purified prior to use according to a modified version of the protocol reported by Boettcher and co-workers.[1] Briefly, centrifuge tubes (50 mL) were cleaned with H_2SO_4. Then, ~2 g of $Ni(NO_3)_2 \cdot 6H_2O$ (99.999 % trace metals basis, Sigma-Aldrich) was dissolved in ~5 mL of Milli-Q water (18 MΩ cm). The active material for purification, $Ni(OH)_2$, was precipitated by additions of ~20 mL of 1 M KOH. In this step, we exchanged the KOH for either LiOH, NaOH, RbOH, or CsOH to match the respective alkali metal cations in the respective hydroxides, and to avoid cross-contaminations of K^+. The solution was then mixed by vigorous shaking, and then centrifuged. The $Ni(OH)_2$ pellet was washed at least three times by additions of ~20 mL Milli-Q and ~1 mL of additional XOH salt. The centrifuge tube containing the washed $Ni(OH)_2$ pellet was then filled with the respective XOH electrolytes, and mixed in a sonicator bath for ~15 min and vigorously shaken, and then let rest overnight. The $Ni(OH)_2$ was then precipitated by centrifugation, and the supernatant was filtered with two H_2SO_4 cleaned Whatman paper filters to remove particles of $Ni(OH)_2$ in the solution. Finally, the 1 M XOH electrolytes were stored in H_2SO_4 cleaned polypropylene bottles until use.

Supplementary Note 2. XAS data processing and EXAFS simulations. The X-ray absorption spectra were processed by averaging several scans (typically between 3-5 scans for each potential). A smoothing function (locally weighted quadratic loss) was employed to reduce the noise in the EXAFS region, while the XANES region including the pre-edge region was left unsmoothed. The oxidation states were obtained from both the edge positions at half-height of the edge jump, and from the M-O coordination distances from simulations using phase functions generated by the FEFF9 code.[2] The simulations were carried out in k-space between 15-295 eV above E_0 (k-range of 2-9 Å^{-1}). The values of E_0 were 8334 eV for the Ni *K*-edge and 7117 eV for the Fe *K*-edge. Due to the narrow k-range in this study, we considered only two shells (M-O and M-M coordination) in the EXAFS simulations. The Debye-Waller parameters (σ) were fixed at reasonable values for the respective shells and the coordination numbers (CN), and absorber-scatterer distances (R) were minimized in the fit. The amplitude reduction factor (S_0^2) was fixed at 0.85 for both Ni and Fe shells. The atom in access in our material (Ni) was selected as the scatterer both at the Ni and Fe *K*-edges, since the difference between using either Ni or Fe phase functions is anyhow not significant within the selected k-range (~1-9 Å^{-1}). The Levenberg-Marquardt algorithm with a 68 % confidence interval was employed. To extract oxidation states, calibration curves were constructed from known reference compounds (Supplementary Fig. 14).

Supplementary Note 3. Alternative approach to determine the experimental pH

A pH calibration curve was established in order to determine the experimental pH in LiOH using the potential difference between the reversible hydroxgen electrode (RHE) and the leak-free Ag/AgCl reference electrode. The calibration curve was constructed from commercial solutions: buffer pH 9 (Certipur®, traceable to SRM from NIST and PTB, Merck), buffer solution pH 12 (Reagecon, traceable to NIST), and commercial KOH solutions; 0.1 M (pH 12.99) and 1 M (pH 13.90) (Titripur®, Supelco). The pH values of commercial KOH solutions were assumed to be the theoretical values determined using the Supplementary Equations (1)-(13), since the concentrations were standardized.

Supplementary Note 4. Calculations of theoretical pH. The pH values were calculated using the tabulated pK_a values (or pK_b values) for hydrated alkali metal cations using the Henderson-Hasselbalch formalism according to the equations (1)-(13) below:

Reactions:

$$[\mathrm{H_2O}] \rightleftarrows [\mathrm{H^+}] + [\mathrm{OH^-}] \tag{1}$$

$$[\mathrm{BOH}] \rightleftarrows [\mathrm{B^+}] + [\mathrm{OH^-}] \tag{2}$$

Definitions:

$$K_\mathrm{b} = \frac{[\mathrm{B^+}][\mathrm{OH^-}]}{[\mathrm{BOH}]} \tag{3}$$

$$pK_\mathrm{b} = -\log_{10} K_\mathrm{b} \tag{4}$$

$$K_\mathrm{w} = [\mathrm{H^+}] \cdot [\mathrm{OH^-}] = 1 \cdot 10^{-14} \tag{5}$$

$$\mathrm{pK_w} = \mathrm{pK_b} + \mathrm{pK_a} = 14 \tag{6}$$

$$\mathrm{pH} = -\log_{10} [\mathrm{H^+}] \tag{7}$$

were brackets indicate a concentration of a species. Then, we express the concentration of [BOH] as the nominal concentration (c) of the hydroxide minus the dissociated [B^+] as follows;

$$[\mathrm{BOH}] = \mathrm{c} - [\mathrm{B^+}] \tag{8}$$

The [BOH] in Equation (3) can now be replaced with the assumption in Equation (8) to obtain following expression:

$$K_\mathrm{b} = \frac{[\mathrm{B^+}] \cdot [\mathrm{OH^-}]}{\mathrm{c} - [\mathrm{B^+}]} \tag{9}$$

Then, charge neutrality is assumed as follows:

$$[\mathrm{B^+}] + [\mathrm{H^+}] = [\mathrm{OH^-}] \tag{10}$$

By combining Equations (5), (9), (10), we will arrive to the following cubic expression below:

$$[\mathrm{H^+}]^3 + \mathrm{c} \cdot [\mathrm{H^+}]^2 - [\mathrm{H^+}] \cdot K_\mathrm{w} - \frac{{K_w}^2}{K_\mathrm{b}} + \frac{[\mathrm{H}]^2 \cdot K_w}{K_\mathrm{b}} = 0 \tag{11}$$

Instead of solving Equation (11) explicitly, it can be solved numerically by finding a value of [H^+] that satisfies the equation.

Since we are dealing with strong bases, we can also assume that the [OH^-] equals the dissociated amount of hydroxide [B^+] in Equation (9), which gives the following quadratic expression which can be solved explicitly:

$$[\mathrm{OH^-}]^2 + K_b \cdot [\mathrm{OH^-}] - K_\mathrm{b} \cdot \mathrm{c} \approx 0 \tag{12}$$

$$[\mathrm{OH^-}] \approx \frac{1}{2}\left(\pm\sqrt{K_\mathrm{b}} \cdot \sqrt{K_\mathrm{b} + 4\mathrm{c}} - K_\mathrm{b}\right) \tag{13}$$

where only the positive solution is relevant. The solution of Equation (13) we confirm is near identical to the solution of Equation (11). The results are presented in Supplementary Table 6.

Supplementary Note 5. Computational methods: DFT-derived reactivity descriptors

Analysis of the bulk and surfaces were conducted using DFT. For the bulk, we estimated the charge distribution by Bader analysis from which we extracted estimated partial charges. For the surfaces, their intrinsic reactivity was estimated by the use of local surface reactivity descriptors. These contain the surface electrostatic potential [$V(\mathrm{r})$], the local average ionization energy [$\bar{I}(\mathrm{r})$],[3] as well as the local electron attachment energy [$E(\mathrm{r})$].[4] These provide a new way of predicating and rationalizing surface reactivity, that has lately been successfully employed to assess the local reactivity of e.g. Cu_2O, TiO_2 and metal surface and nanoparticle.[5,6,7] These are defined at the position r in space according to Equations (14)-(16):

$$\mathrm{V}(\mathbf{r}) = \sum_{\mathrm{A}} \frac{\mathrm{Z_A}}{|\mathbf{R}_\mathrm{A} - \mathbf{r}|} - \int \frac{\rho(\mathbf{r}')\mathrm{d}\mathbf{r}'}{|\mathbf{r}' - \mathbf{r}|} \tag{14}$$

$$\bar{\mathrm{I}}(\mathbf{r}) = -\sum_{\mathrm{i}=1}^{\mathrm{E_F}} \frac{\varepsilon_\mathrm{i}\rho_\mathrm{i}(\mathbf{r})}{\rho(\mathbf{r})} \tag{15}$$

$$\mathrm{E}(\mathbf{r}) = \sum_{\varepsilon_\mathrm{i}=\mathrm{E_F}}^{\varepsilon_\mathrm{i}<0} \frac{\varepsilon_\mathrm{i}\rho_\mathrm{i}(\mathbf{r})}{\rho(\mathbf{r})} \tag{16}$$

where Z_A and R_A are the charge and position of the A:th nuclei, $\rho(\mathrm{r})$ the total electron density function, while $\rho_i(\mathrm{r})$ and ε_i are the density function and eigenvalue of the i:th Kohn-Sham spin states. The sum over states run over the occupied states up to the Fermi level (E_F) for $\bar{I}(\mathrm{r})$ and over the unoccupied states (below the free electron limit, $\varepsilon_i > 0$) for $E(\mathrm{r})$. Accordingly, $\bar{I}(\mathrm{r})$ describes a compound's local electron-donating abilities (i.e. nucleophilicity/Lewis basicity), while $E(\mathrm{r})$ reflects the its local electron accepting capacity (i.e. electrophilicity/Lewis acidity). $V(\mathrm{r})$ can identify both Lewis acidic (positive regions) and Lewis basic (negative regions) sites. Whereas $V(\mathrm{r})$ reflects the electrostatic interaction tendencies of the compound, $\bar{I}(\mathrm{r})$ and $E(\mathrm{r})$ also describes the its charge-transfer/polarization capabilities. Hence these properties are largely complementary. They also capture the terms that are usually of most importance in an interaction – electrostatics and charge-transfer/polarization. These properties are evaluated on the 0.001 au isodensity surface contour. Local extreme points of the surface properties (indexed with S) can be used to identify reactive sites or interaction sites – $V_{S,\mathrm{max}}$ and $E_{S,\mathrm{min}}$ identifies Lewis acidic sites while $V_{S,\mathrm{min}}$ and $\bar{I}_{S,\mathrm{min}}$ identifies Lewis basic sites. Excellent correlations between the magnitude of the extreme points and the local adsorption enthalpies have been found for a range of compounds including molecules, nanoparticles and surfaces of metals and semiconductors. [5]

The Ni/Fe/O sites on the (100) surface model is inequivalent. Supplementary Fig. 21 vide infra show which sites have been included in the reactivity property analysis. For comparison, the marked Ni sites where exchanged for Fe and the properties revaluated for the case of the all cations.

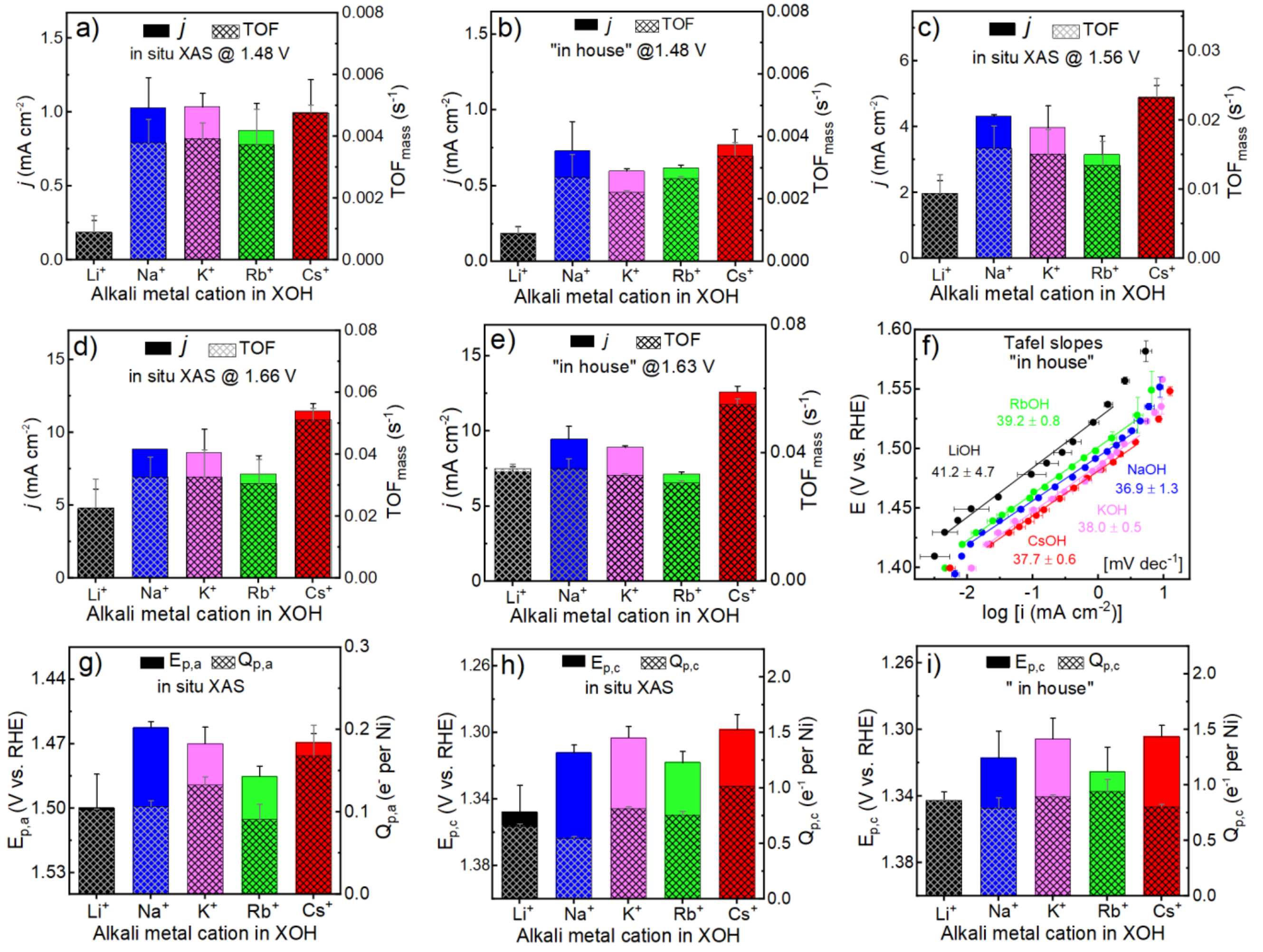

Supplementary Fig. 1. OER activity and redox peak features of the $Ni_{65}Fe_{35}$ catalyst in purified 0.1 M XOH electrolytes (X = Li^+, Na^+, K^+, Rb^+, Cs^+) **(a)** Geometric current density (*j*) at 1.48 V (solid, left) and the TOF_{mass} based on both the Ni and Fe content on the electrode (hatched bars, right), obtained during the collection of the *K*-edges at the P64 beamline at Petra III, which is denoted "in situ XAS". **(b)** *j* at 1.48 V (left) and TOF_{mass} (right), obtained from a larger data set measured "in house", also during steady-state conditions. **(c)** *j* at 1.56 V (left) and TOF_{mass} (right) from in situ XAS. **(d)** *j* at 1.66 V (left) and TOF_{mass} (right) from in situ XAS. **(e)** *j* at 1.63 V (left) and TOF_{mass} (right) measured "in house". **(f)** Tafel slopes from steady-state conditions measured "in house". **(g)** Anodic peak position, $E_{p,a}$ (left) and the integrated peak charge, $Q_{p,a}$ (right) of the $Ni^{2+} \rightarrow Ni^{(3+\delta)+}$ redox peak from in situ XAS. **(h)** The $E_{p,c}$ (left) and $Q_{p,c}$ (right) obtained from in situ XAS. **(i)** The $E_{p,c}$ (left) and $Q_{p,c}$ (right) measured "in house". The in situ XAS data was measured using a leak-free Ag/AgCl reference electrode, and the "in house" data using a reversible hydrogen electrode (RHE). All data is reported against the RHE reference electrode. All peak potentials (E_p) and peak charges (Q_p) were extracted from CVs at a scan-rate of 10 mV s^{-1}, whereas the OER activity data is obtained from steady-state conditions. Error bars represent the standard error from the overall number of measurements.

Supplementary Table 1. OER activity parameters of the $Ni_{65}Fe_{35}$ catalyst in 0.1 M purified alkali hydroxides (LiOH, NaOH, KOH, RbOH, CsOH). The TOF values were obtained from the data collected from the potential steps at the occasion of the in situ XAS measurements at Petra III (DESY) (measured using a leak-free Ag/AgCl reference electrode), whereas the overpotential (η_{OER}) at 10 mA cm^{-2} and Tafel slopes were measured "in house" using a reversible hydrogen electrode (RHE). All values are reported on the RHE scale. The turnover frequency (TOF_{mass}) accounts for both Ni and Fe metal. The uncertainty of the last digit(s) is given in parenthesis, and shows the standard error.

Electrolyte (0.1 M)	Cation radius (pm) [(a)]	iR-drop (Ω)	Loading (ICP) (μg cm^{-2}) [(b)]	*j* @1.48V (mA cm^{-2})	TOF @1.48 V (s^{-1})	*i* @1.56 V (mA cm^{-2})	TOF@1.56 V (s^{-1})	η_{OER} @ 10 mA cm^{-2} (mV)	Tafel slope (mV dec^{-1})
LiOH	69	36 (13)	21 (4)	0.2 (1)	0.009 (3)	2.0 (4)	0.009 (3)	352 (20)	41 (5)
NaOH	102	31 (12)	27 (1)	1.0 (2)	0.016 (3)	4.3 (1)	0.016 (3)	309 (2)	37 (1)
KOH	138	31 (11)	26 (1)	1.0 (1)	0.015 (4)	4.0 (7)	0.015 (4)	316 (17)	38 (1)
RbOH	149	31 (16)	23 (2)	0.9 (2)	0.014 (4)	3.1 (6)	0.014 (4)	331 (27)	39 (1)
CsOH	170	33 (10)	21 (2)	1.0 (2)	0.023 (3)	4.9 (4)	0.023 (3)	295 (5)	38 (1)

[(a)] The cation radius was obtained from Marcus et al.[8]

[(b)] The Ni:Fe compositions and loadings were determined after the electrochemical characterization using ICP-OES.

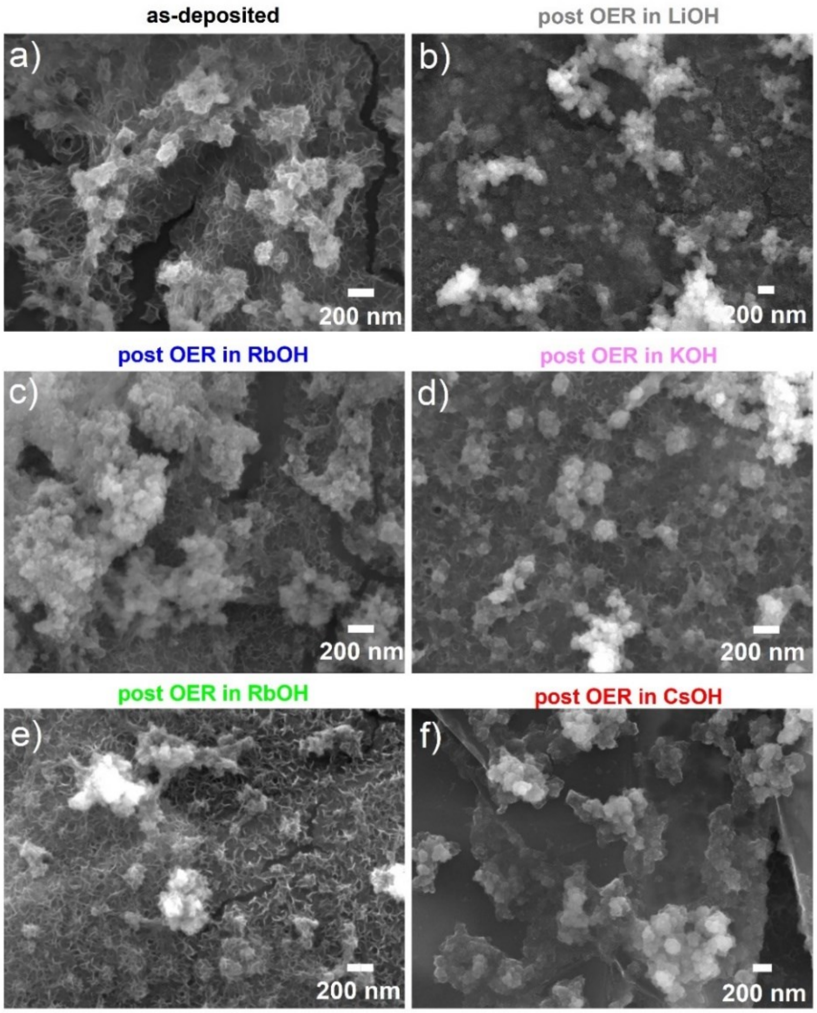

Supplementary Fig. 2. Scanning Electron Microscopy (SEM) of the electrodeposited $Ni_{65}Fe_{35}$ catalyst on graphene working electrodes **(a)** as-deposited film not exposed to electrolyte, and films investigated post-OER after the in situ XAS measurements in purified 0.1 M alkali hydroxides; **(b)** LiOH **(c)** NaOH **(d)** KOH **(e)** RbOH and **(f)** CsOH. The electrodes were rinsed briefly with Milli-Q water before analyzed.

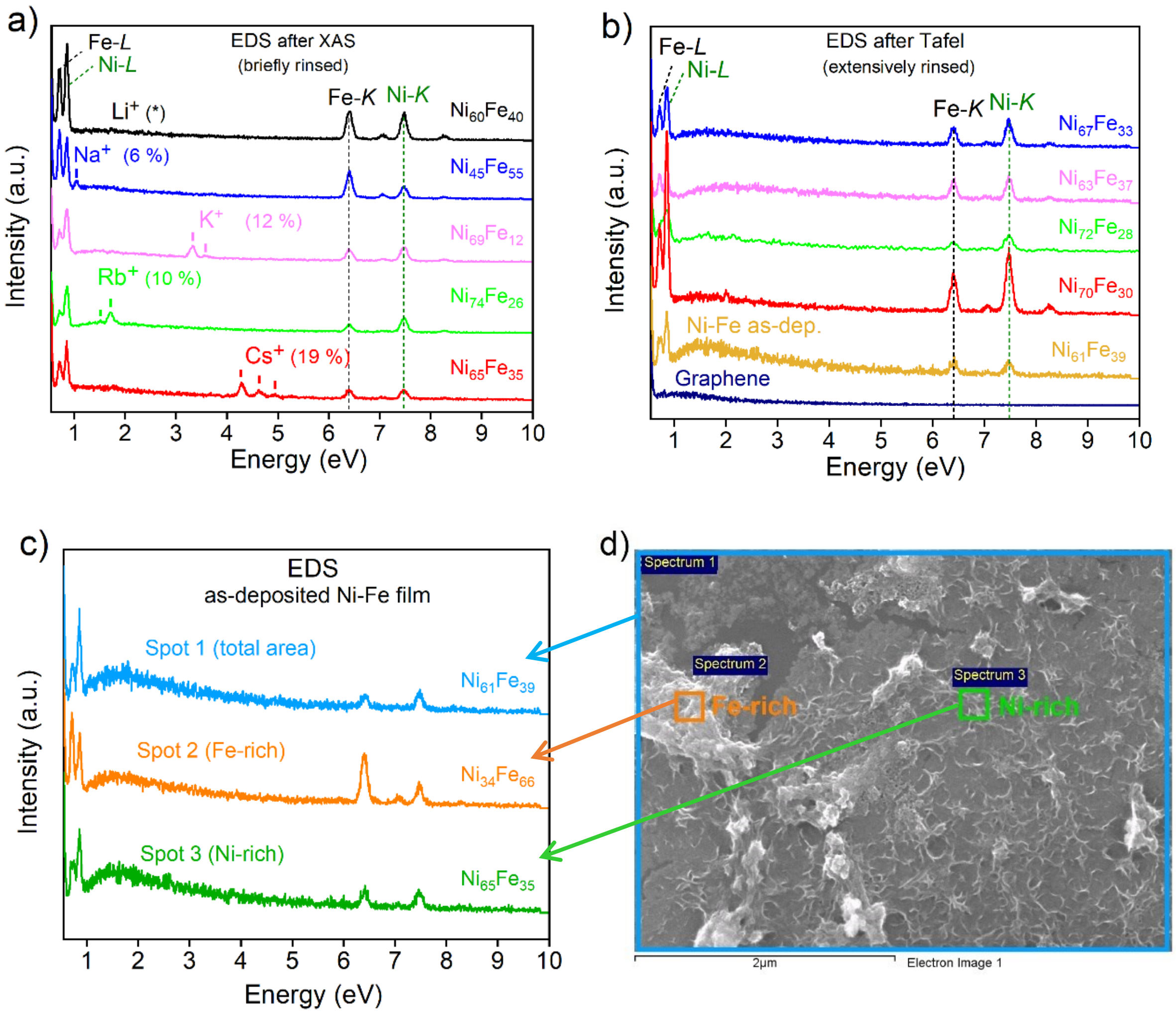

Supplementary Fig. 3. Energy dispersive X-ray spectroscopy (EDS) of the $Ni_{65}Fe_{35}$ films on graphene electrodes after investigations in 0.1 M LiOH, NaOH, KOH, RbOH, CsOH. **(a)** Selected EDS spectra of sample areas after the in situ XAS measurements. These samples were rinsed only briefly with Milli-Q water after the measurements (i.e. the rinsing step was not controlled), and traces of alkali metal cations were visible in the films in the EDS spectra, marked with drop-lines. The atomic percentages (at. %) are given in brackets. **(b)** EDS spectra of selected sample areas after Tafel slope collection. These samples were rinsed extensively (i.e. in a controlled manner) by dipping the films five times in fresh Milli-Q water immediately after the measurements to remove non-specifically adsorbed salts before drying. There were no traces of alkali metal cations detected in these films. The two lower EDS spectra show an as-deposited Ni-Fe film (yellow), and an empty graphene electrode (dark blue). **(c)** Selected EDS spectra demonstrating Ni-rich and Fe-rich areas in the as-deposited Ni-Fe catalyst film. Areas with compositional inhomogeneity were also visible post-OER (not shown here). **(d)** The corresponding SEM image of the respective Ni-rich and Fe-rich areas shown in (c). *) Li^{+} is a too light element to be detected with EDS.

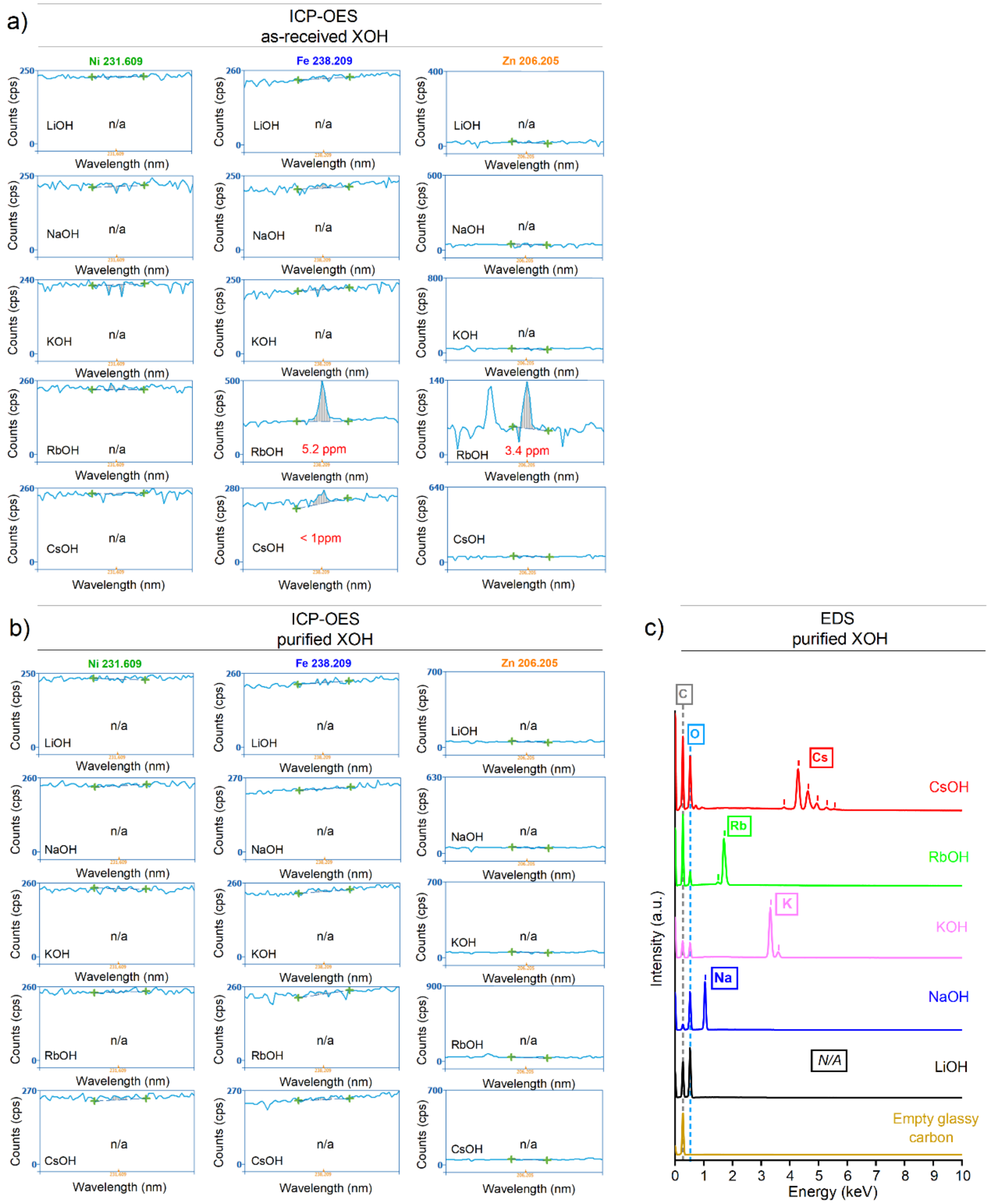

Supplementary Fig. 4. Elemental analysis of the alkali hydroxides addressing impurities. All electrolytes had been diluted to a final concentration of 1000 mg/L (ppm) with respect to the cation. **(a)** as-received alkali hydroxides **(b)** purified alkali hydroxides (XOH). The as-received RbOH shows ~5 ppm Fe and ~3 ppm Zn impurities. In CsOH, there is only slightly elevated levels of Fe, approximately ≤ 1 ppm, however, is a bit uncertain considering the noise level. **(c)** EDS spectra of 1 M purified XOH, dried on (H_2SO_4 cleaned) glassy carbon plates (4 x 4 mm, SIGRADUR® K, HTW) using the build-in EDS detector of the Zeiss LEO1550 microscope. Elemental maps were recorded for ~10-15 min at 2-3 different areas (300 x 200 µm spot size), using an acceleration voltage of 15 kV. The variations in the carbon content in the EDS spectra depends on how homogenous the distributions of the dried salts are, and has no significance. We do also not interpret the oxygen content since the accuracy is low for soft elements, but is on average 66 ± 10 at. % for the hydroxides, of which ~3 at. % originates from the empty glassy carbon plate. It should be noted that Li^+ is too light to be detected in EDS, and Cs^+ could not be detected using ICP-OES due to the too low emission intensity.

Supplementary Table 2. ICP-OES and EDS analysis of the $Ni_{65}Fe_{35}$ catalysts before and after OER in 0.1 M XOH (X = Li^+, Na^+, K^+, Rb^+, and Cs^+) electrolytes. The elemental compositions were determined using energy dispersive X-ray spectroscopy (EDS) and ICP-OES after the in situ XAS measurements and Tafel slope collection. The films were rinsed either briefly in an uncontrolled manner, or carefully in a more controlled manner using Milli-Q water. All elemental compositions are reported in atomic %, and the metal loadings in weight %. The numbers reported in this table are the averages of several investigated areas or films.

Material analyzed	Exposed to electrolyte:	Method	Films analyzed after:	Rinsing protocol	Elemental composition (at. %)						Metal loading ($\mu g\ cm^{-2}$)	
EDS analysis (bulk; local)					Ni-rich area (at. %)			Fe-rich area (at %)			Local	
					Ni	Fe	X	Ni	Fe	X	Ni	Fe
$Ni_{65}Fe_{35}$	as-deposited	EDS	n/a[(a)]	n/a[(a)]	66	34	0	34	66	0	n/a[(a)]	n/a[(a)]
$Ni_{65}Fe_{35}$	LiOH	EDS	XAS	Brief [(b)]	64	36	n/a[(d)]	54	46	n/a[(d)]	n/a[(a)]	n/a[(a)]
$Ni_{65}Fe_{35}$	NaOH	EDS	XAS	Brief [(b)]	66	25	9	18	70	12	n/a[(a)]	n/a[(a)]
$Ni_{65}Fe_{35}$	KOH	EDS	XAS	Brief [(b)]	60	29	11	35	43	22	n/a[(a)]	n/a[(a)]
$Ni_{65}Fe_{35}$	RbOH	EDS	XAS	Brief [(b)]	68	24	8	32	50	18	n/a[(a)]	n/a[(a)]
$Ni_{65}Fe_{35}$	CsOH	EDS	XAS	Brief [(b)]	57	24	18	20	69	10	n/a[(a)]	n/a[(a)]
$Ni_{65}Fe_{35}$	NaOH	EDS	Tafel	Extensive [(c)]	70	31	0	n/a[(f)]	n/a[(f)]	n/a[(f)]	n/a[(a)]	n/a[(a)]
$Ni_{65}Fe_{35}$	KOH	EDS	Tafel	Extensive [(c)]	71	29	0	n/a[(f)]	n/a[(f)]	n/a[(f)]	n/a[(a)]	n/a[(a)]
$Ni_{65}Fe_{35}$	CsOH	EDS	Tafel	Extensive [(c)]	74	26	0	n/a[(f)]	n/a[(f)]	n/a[(f)]	n/a[(a)]	n/a[(a)]
ICP-OES analysis (bulk; average entire film)					Average (at. %)			Average (at. %)			Average	
					Ni	Fe	X	Ni:Fe			Ni	Fe
$Ni_{65}Fe_{35}$	LiOH	ICP-OES	XAS	Brief [(a)]	32	19	50	63:37			13	8
$Ni_{65}Fe_{35}$	NaOH	ICP-OES	XAS	Brief [(a)]	51	22	26	70:30			19	8
$Ni_{65}Fe_{35}$	KOH	ICP-OES	XAS	Brief [(a)]	61	36	2	62:38			17	10
$Ni_{65}Fe_{35}$	RbOH	ICP-OES	XAS	Brief [(a)]	25	16	59	60:40			12	8
$Ni_{65}Fe_{35}$	CsOH	ICP-OES	XAS	Brief [(a)]	67	33	n/a[(e)]	67:33			16	7

(a) Does not apply

(b) The rinsing protocol was not carried out in a controlled manner, so either the films were rinsed briefly or not washed at all before analyzed.

(c) The samples were rinsed in a controlled manner, where the films were dipped five times in fresh Milli-Q water, and afterwards dried with N_2 gas before analyzed.

(d) Li^+ cannot be detected by EDS (too light element)

(e) Cs^+ could not be determined by ICP-OES

(f) Not investigated

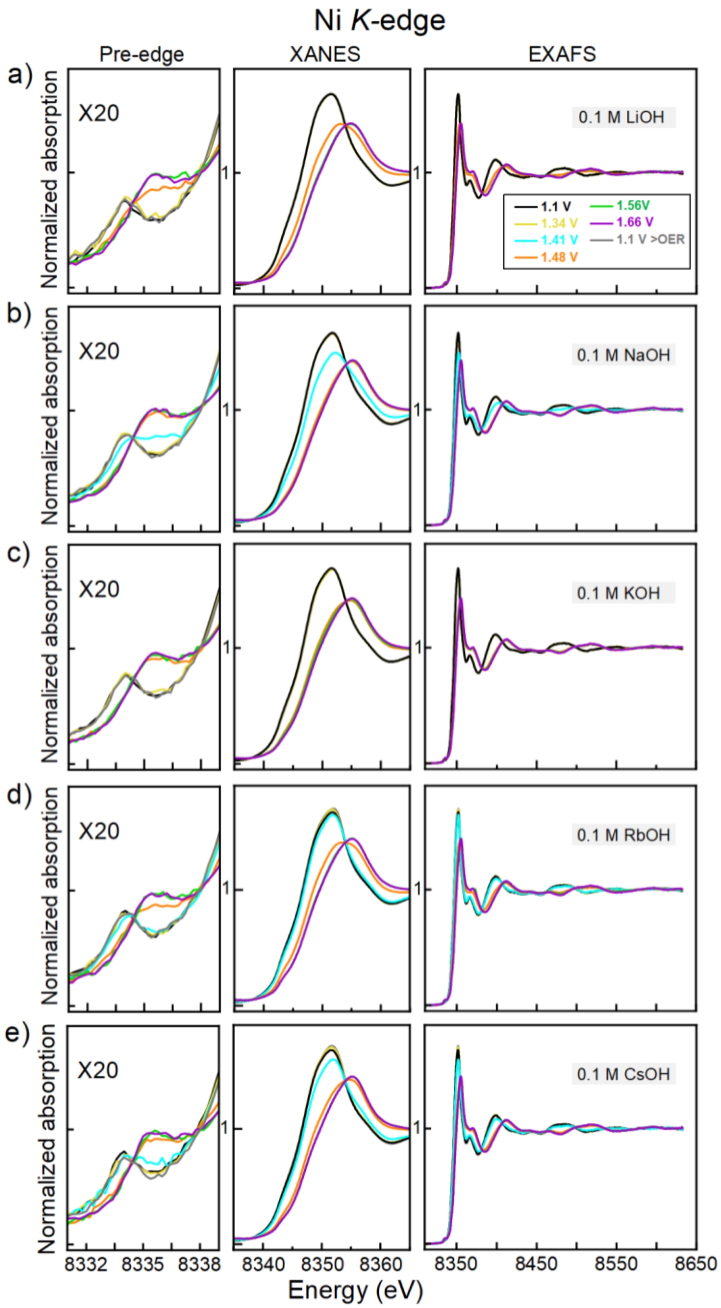

Supplementary Fig. 5. In situ XAS at the Ni *K*-edge of the $Ni_{65}Fe_{35}$ catalyst at different potentials in purified 0.1 M alkali hydroxides. Shown is the $k^3(k)$ pre-edge region (left figure), the XANES region (middle figure), and the EXAFS region (right figure) in **(a)** LiOH **(b)** NaOH, **(c)** KOH, **(d)** RbOH, **(e)** CsOH. The legend in (a) applies to all spectra. The data was recorded at the P64 beamline at Petra III (DESY). The Y-axis of the pre-edge (left figure) is enlarged 20 times with respect to the XANES (middle) and EXAFS region (right).

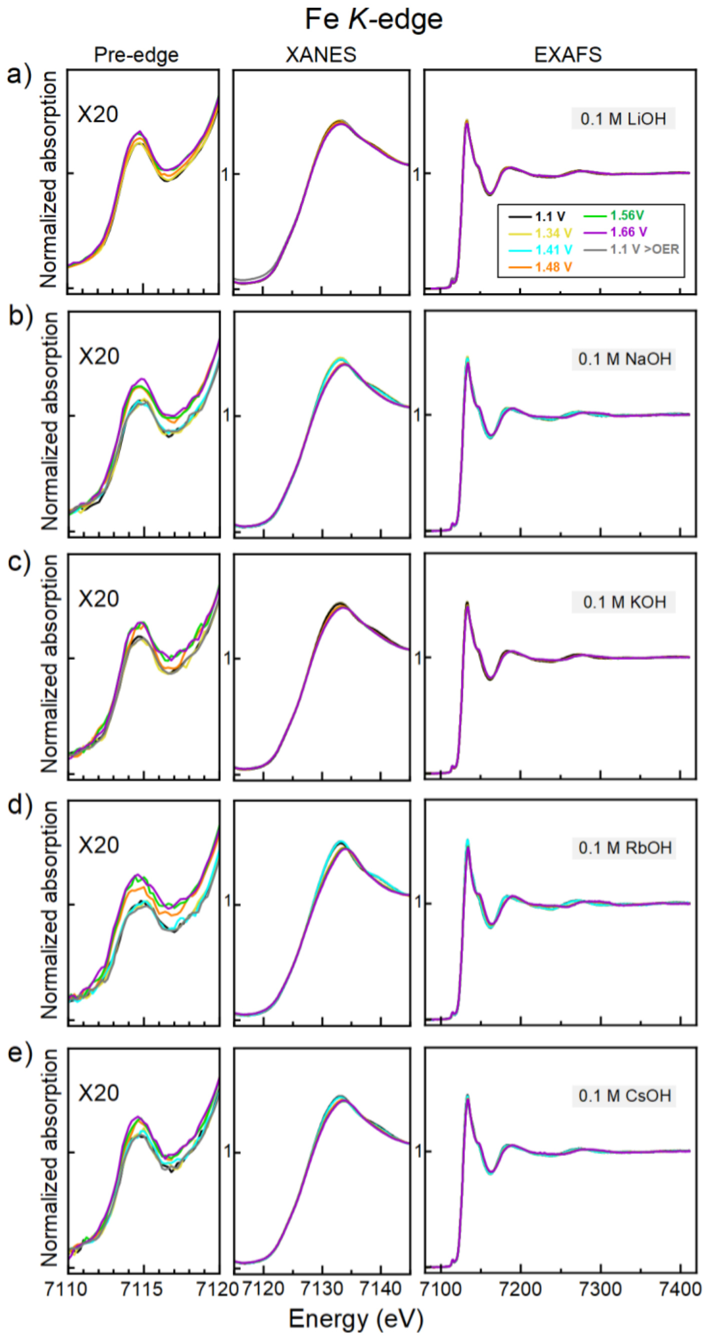

Supplementary Fig. 6. In situ XAS at the Fe *K*-edge of the $Ni_{65}Fe_{35}$ catalyst at different potentials in purified 0.1 M alkali hydroxides. Shown is the $k^3(k)$ pre-edge region (left figure), the XANES region (middle figure), and the EXAFS region (right figure) in **(a)** LiOH **(b)** NaOH, **(c)** KOH, **(d)** RbOH, **(e)** CsOH. The legend in (a) applies to all spectra. The data is from the P64 beamline at Petra III (DESY). The Y-axis of the pre-edge (left figure) is enlarged 20 times with respect to the XANES (middle) and EXAFS region (right).

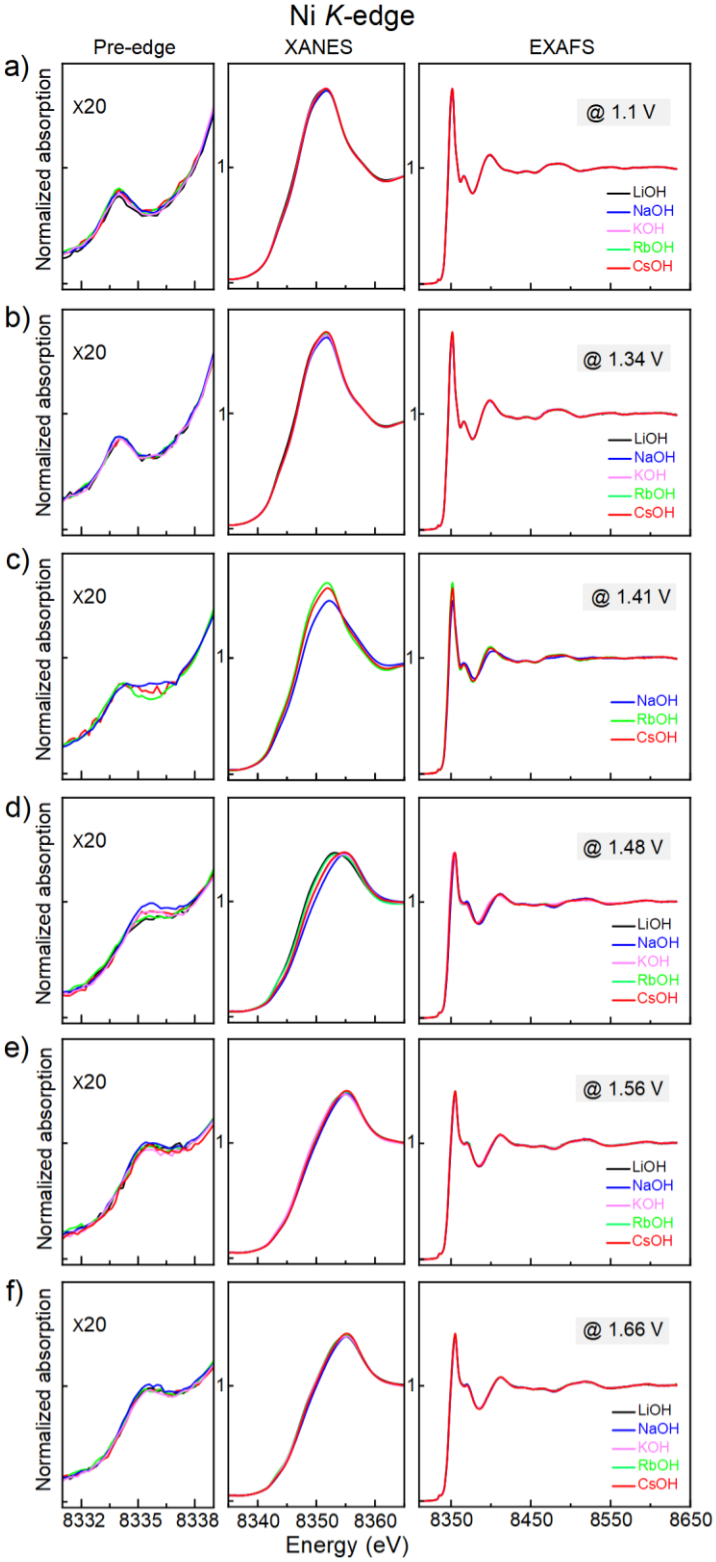

Supplementary Fig. 7. In situ XAS at the Ni *K*-edge of the $Ni_{65}Fe_{35}$ catalyst in different purified 0.1 M alkali hydroxides (LiOH, NaOH, KOH, RbOH, CsOH). Shown is the $k^3(k)$ pre-edge region (left figure), the XANES region (middle figure), and the EXAFS region (right figure) at **(a)** 1.1 V **(b)** 1.34 V **(c)** 1.41 V **(d)** 1.48 V **(e)** 1.56 V, and **(f)** 1.66 V vs. RHE. The data is from the P64 beamline at Petra III (DESY). The Y-axis of the pre-edge (left figure) is enlarged 20 times with respect to the XANES (middle) and EXAFS region (right).

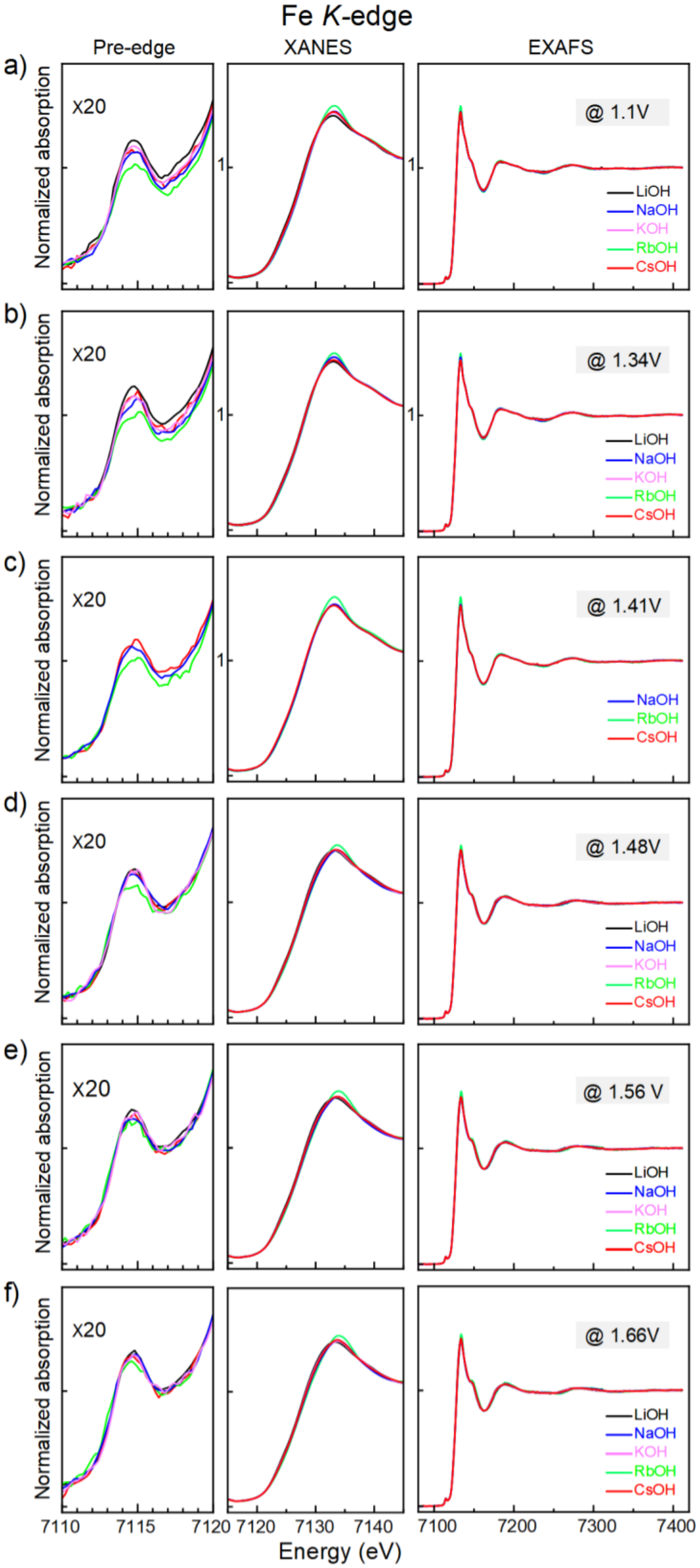

Supplementary Fig. 8. In situ XAS at the Fe *K*-edge of the $Ni_{65}Fe_{35}$ catalyst in different purified 0.1 M alkali hydroxides (LiOH, NaOH, KOH, RbOH, CsOH). Shown is the $k^3(k)$ pre-edge region (left figure), the XANES region (middle figure), and the EXAFS region (right figure) at **(a)** 1.1 V **(b)** 1.34 V **(c)** 1.41 V **(d)** 1.48 V **(e)** 1.56 V, and **(f)** 1.66 V vs. RHE. The data is from the P64 beamline at Petra III (DESY). The Y-axis of the pre-edge (left figure) is enlarged 20 times with respect to the XANES (middle) and EXAFS region (right).

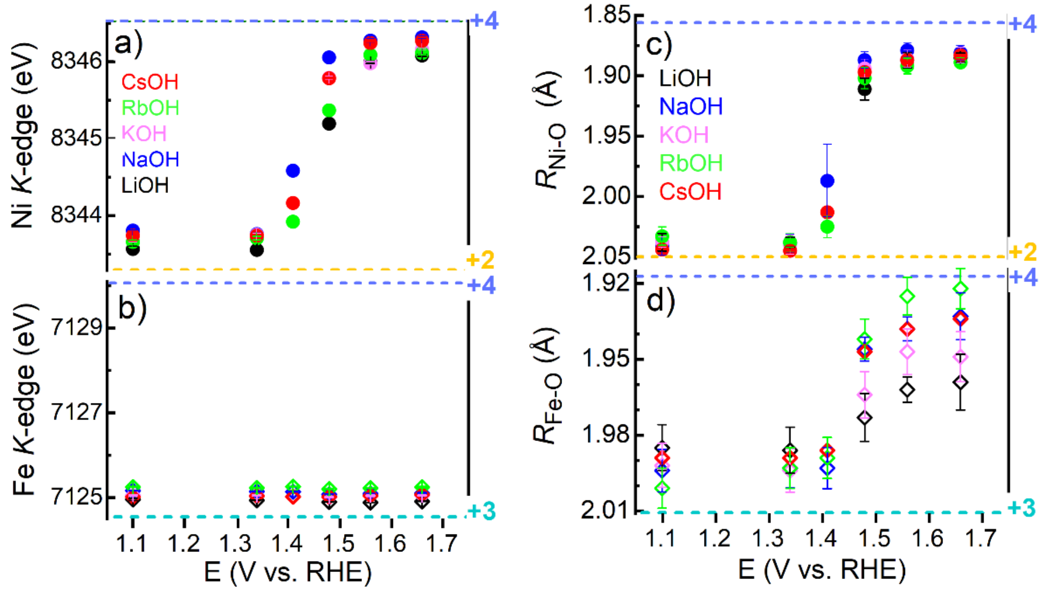

Supplementary Fig. 9. XAS trend plots against the applied potential. Trends from in situ XAS at the metal *K*-edges of the electrodeposited $Ni_{65}Fe_{35}$ catalyst in purified 0.1 M XOH electrolytes (X= Li^+, Na^+, K^+, Rb^+, Cs^+) at different electrode potentials; 1.1 V, 1.34 V, 1.41 V, 1.48 V, 1.56 V, 1.66 V vs. RHE. **(a)** The Ni *K*-edge positions. **(b)** The Fe *K*-edge positions. **(c)** The Ni-O coordination distances obtained by simulations of the $k^3(k)$ EXAFS spectra. **(d)** The corresponding Fe-O coordination distances. The coloured dashed lines at the top and bottom indicate the oxidation states; +2 (yellow), +3 (green), +4 (purple). The shown data is from the P64 beamline at Petra III (DESY).

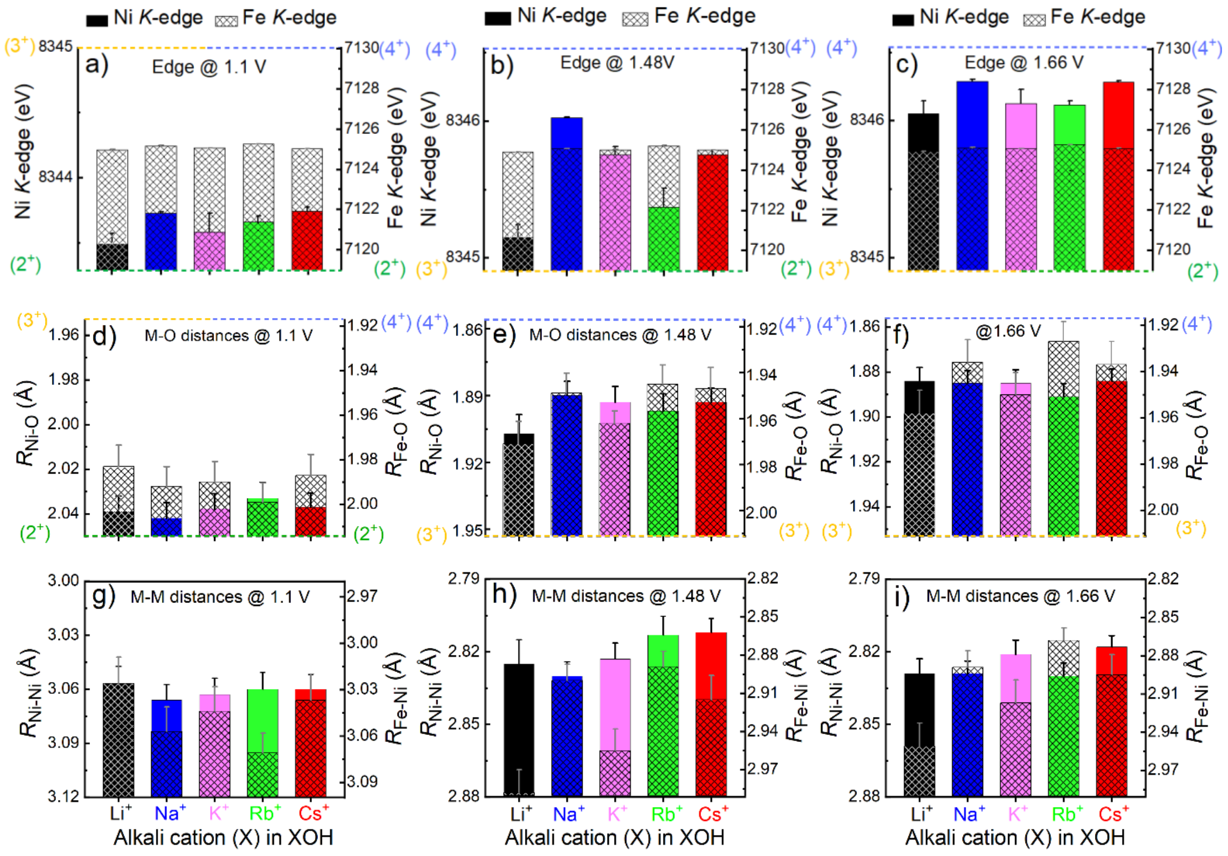

Supplementary Fig. 10. XAS trend plots. The *K* edge positions and coordination distances are shown at various electrode potentials in purified 0.1 M XOH electrolytes (X= Li^+, Na^+, K^+, Rb^+, Cs^+). **(a)** *K*-edge positions at 1.1 V (not oxidized, non-catalytic) **(b)** *K*-edge positions at 1.48 V (half-oxidized state, low catalytic rate) **(c)** *K*-edge positions at 1.66 V (near fully oxidized state, high catalytic rate). **(d)** M-O coordination distances at 1.1 V **(e)** M-O coordination distances 1.48 V **(f)** M-O coordination distances 1.66 V. **(g)** M-M coordination distances at 1.1 V **(h)** M-M coordination distances at 1.48 V **(i)** M-M coordination distances at 1.66 V. Potentials were recorded using a leak-free Ag/AgCl reference electrode, however, is reported against RHE. The Ni *K*-edge is shown on the left axis (filled bars), and the Fe *K*-edge on the right axis (hatched bars). The dashed lines at the top and bottom indicate the oxidation states; +2 (green), +3 (yellow), +4 (purple). The data is from the P64 beamline at Petra III (DESY). Error bars represent the standard error from the overall number of measurements.

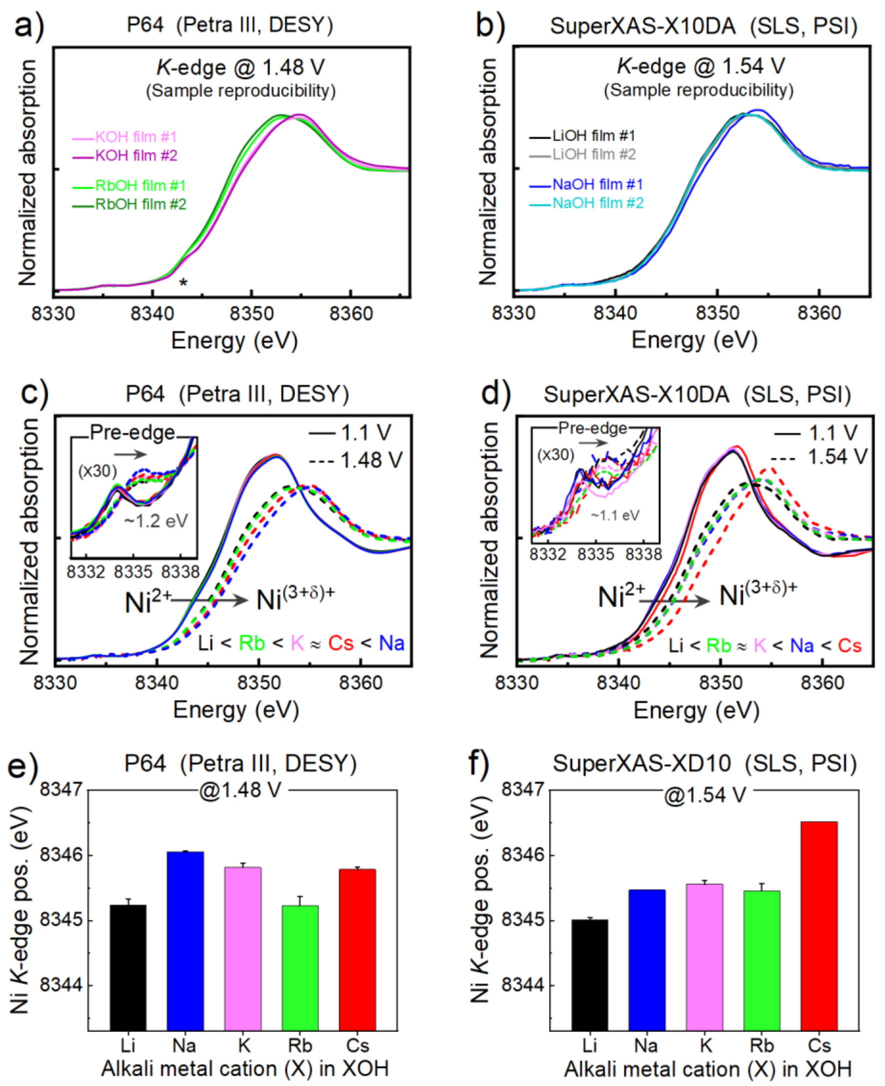

Supplementary Fig. 11. Comparison of the Ni *K*-edge positions from different beamlines (P64, Petra III, DESY and SuperXAS S10DA - SLS, PSI) **(a)** Ni *K*-edges of the $Ni_{65}Fe_{35}$ catalyst at 1.48 V from P64-Petra III, demonstrating reproducibility for different films. The catalyst shown had a metal loading of ~25 ± 2 µg cm^{-2}, corresponding to a film thickness of ~300 nm. **(b)** Ni *K*-edges of a $Ni_{75}Fe_{25}$ catalyst at 1.54 V from SuperXAS-SLS, showing reproducibility of different films. This catalyst had a metal loading of 3 ± 1 µg cm^{-2}, and a film thickness of ~50 nm. **(c)** The Ni *K*-edges at 1.48 V in different alkali hydroxides measured at P64-Petra III. The inset shows an enlargement of the pre-edge region. **(d)** The Ni *K*-edges at 1.54 V in the different alkali hydroxides measured at SuperXAS-SLS. The inset shows an enlargement of the pre-edge region. **(e)** Trend plot of the Ni *K*-edge positions measured at P64 -Petra III. **(f)** Trend plot of the Ni *K*-edge positions from SuperXAS-SLS. The selected potentials (1.48 V vs. 1.54 V, respectively) are selected based on the absolute *K*-edge shifts, since these were different for the two beamtimes because of different film thicknesses (~300 nm at Petra III versus ~50 nm at SLS). Note that a fresh catalyst film was used for each new cation/hydroxide at Petra III, whereas the same film was recycled for different cations at SuperXAS-SLS using a “cation switching” protocol. The films were rinsed with plenty of Milli-Q water in between the switching. The star (*) in (a) marks a glitch in the monochromator crystal that is sometimes visible if not perfectly cancelled by the I_0 signal. Error bars represent the standard error from the overall number of measurements.

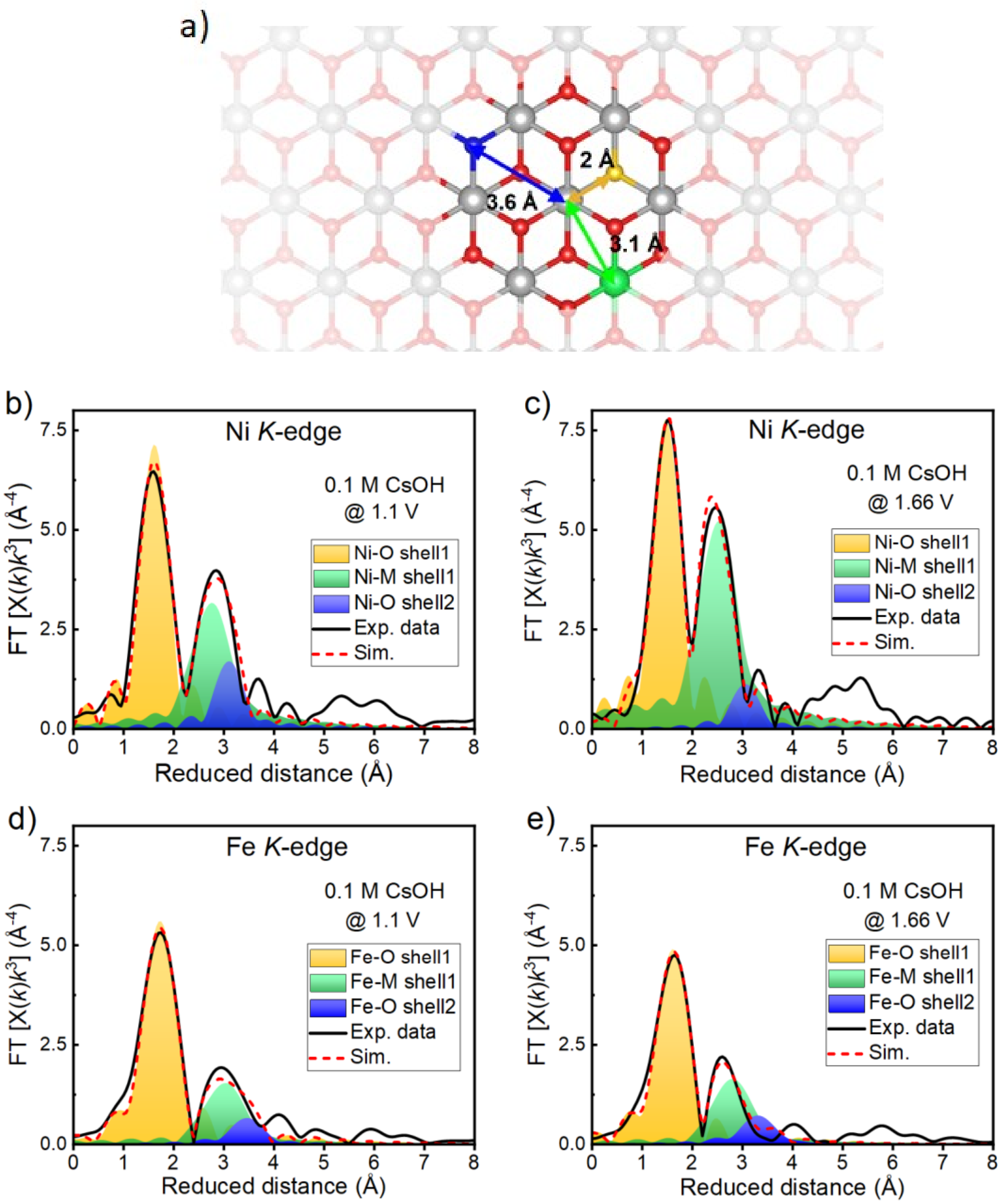

Supplementary Fig. 12. EXAFS simulation using scattering functions. Simulations of the $k^3(k)$ EXAFS spectra of the $Ni_{65}Fe_{35}$ catalyst in the FT-EXAFS domain in 0.1 M CsOH as an example of the simulation approach. Scattering functions were generated in FEFF9 with the self-consistent field option switched on.[1] **(a)** Schematic representation of the atomic coordination distances that were considered in the simulations using α-$Ni(OH)_2$ as example; the nearest Ni-O distance (yellow), nearest Ni-Ni distance (green), and 2nd nearest Ni-O distance (blue). **(b)** The Ni *K*-edge at 1.1 V. **(c)** The Ni *K*-edge at 1.66 V. **(d)** The Fe *K*-edge at 1.1 V. **(e)** The Fe *K*-edge at 1.66 V. The different components (shells) considered in the simulation are shown as colored areas under the peaks according to the colour code given in (a). The black curves are the experimental data and the dashed red black curves the sum of the simulated scattering shells. The data shown here is from the P64 beamline at Petra III (DESY).

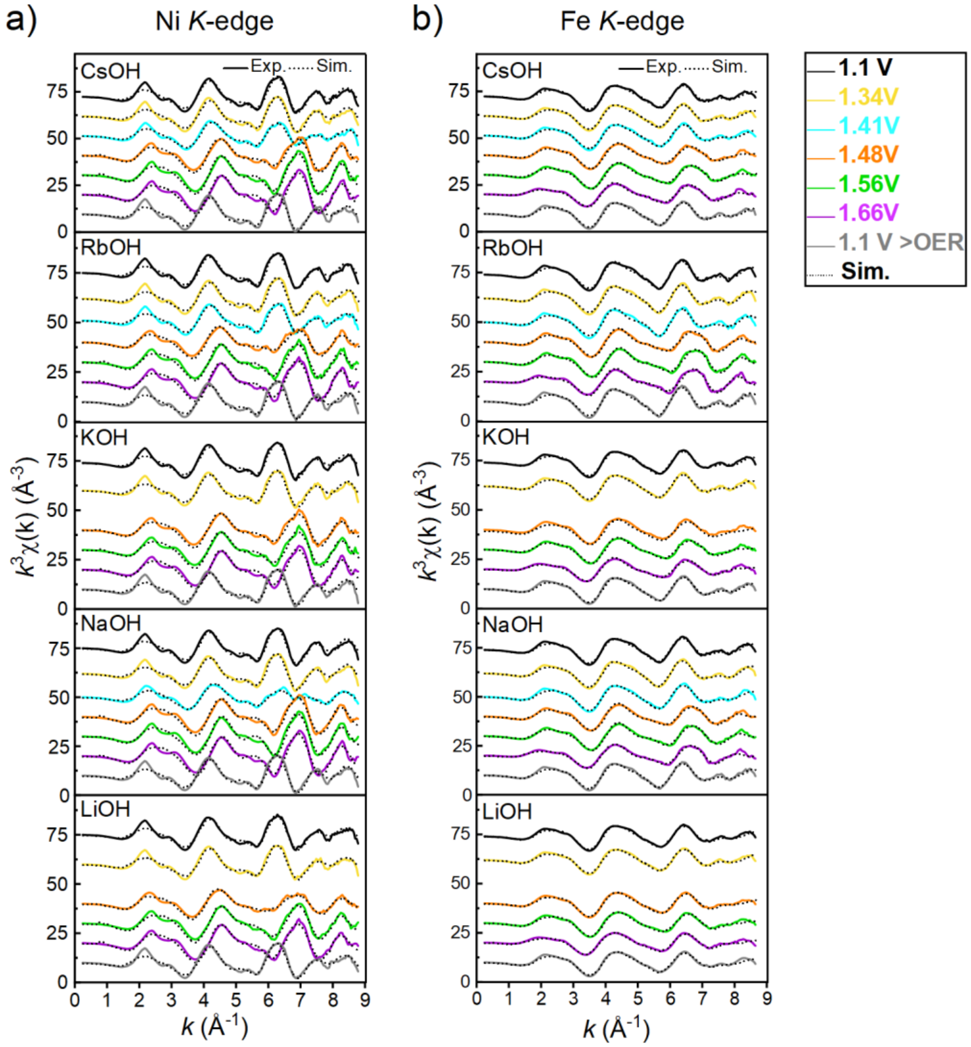

Supplementary Fig. 13. The $k^3(k)$ EXAFS oscillations of the $Ni_{65}Fe_{35}(OOH)$ catalyst. **(a)** Ni *K*-edge **(b)** Fe *K*-edge. The measurements were performed in purified alkali metal hydroxides (0.1 M CsOH, RbOH, KOH, NaOH, LiOH) at different electrode potentials (1.1 V, 1.31 V, 1.34, 1.41, 1.48, 1.56, and 1.66 V vs. RHE). The coloured curves are the experimental data and the dotted black curves the simulated spectra using FEFF scattering functions. The legend to the upper right applies to all spectra. The data shown here is from the P64 beamline at Petra III (DESY).

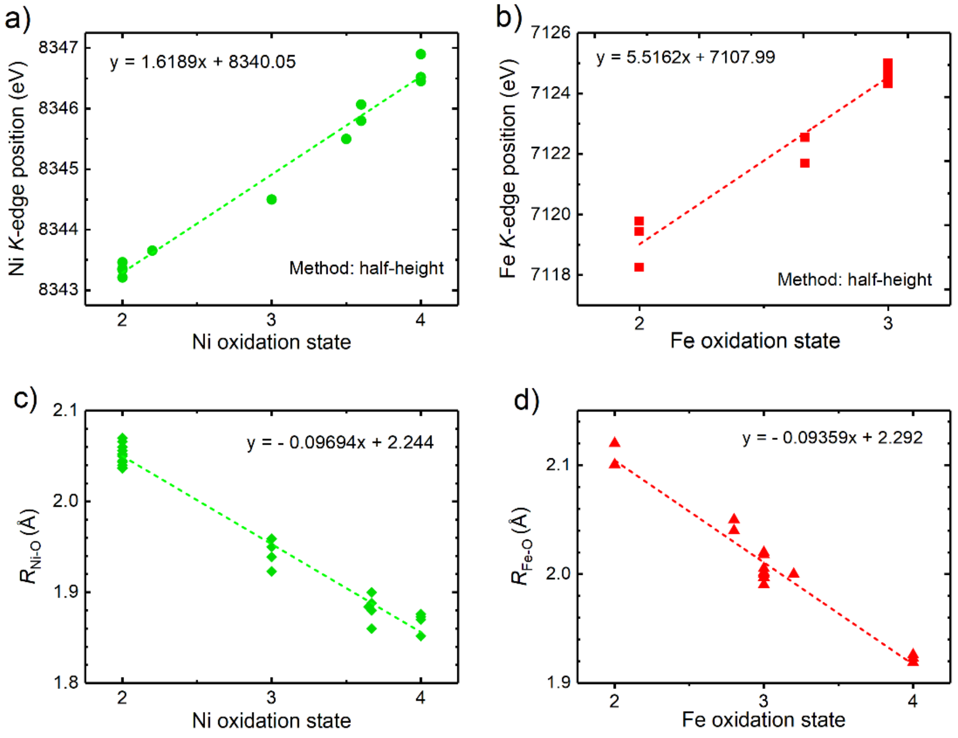

Supplementary Fig. 14. XAS calibration curves for the metal oxidation states and atomic coordination distances obtained from known reference compounds at the Ni and Fe *K*-edges. The references are both measured by us, and taken from literature.[9–12] **(a)** Ni *K*-edge positions at half-height of the normalized edge-jump. **(b)** Fe *K*-edge positions at half-height of the normalized edge-jump. **(c)** Ni-O coordination distances ($R_{Ni\text{-}O}$). **(d)** Fe-O coordination distances ($R_{Fe\text{-}O}$), plotted against the oxidation state.

Supplementary Table 3. Ni and Fe *K*-edge positions of the $Ni_{65}Fe_{35}(OOH)$ catalyst in 0.1 M alkali hydroxides at different potentials during in situ XAS, measured at the P64 beamline at Petra III (DESY). The *K*-edge positions were extracted at half-height of the normalized edge jump, and the oxidation states were obtained from calibration curves in Supplementary Fig. 14a-b. The uncertainty of the last digit(s) is given in parenthesis, and shows the standard error.

	Ni *K*-edge		Fe *K*-edge	
$Ni_{65}Fe_{35}$	Ni *K*-edge (eV)	Ni ox. state (edge pos.)	Fe *K*-edge (eV)	Fe ox. state (edge pos.)
E = 1.1 V				
LiOH	8343.5 (1)	2.12 (5)	7125.0 (1)	3.08 (1)
NaOH	8343.7 (1)	2.27 (6)	7125.2 (1)	3.11 (1)
KOH	8343.6 (2)	2.18 (9)	7125.1 (1)	3.09 (1)
RbOH	8343.7 (1)	2.23 (3)	7125.2 (3)	3.13 (5)
CsOH	8343.7 (1)	2.28 (6)	7125.0 (1)	3.08 (1)
E = 1.34 V				
LiOH	8343.5 (1)	2.11 (2)	7124.9 (3)	3.07 (1)
NaOH	8343.7 (1)	2.24 (6)	7125.1 (1)	3.11 (1)
KOH	8343.7 (2)	2.24 (12)	7125.0 (1)	3.09 (1)
RbOH	8343.7 (1)	3.26 (6)	7125.2 (3)	3.13 (5)
CsOH	8343.7 (1)	2.28 (4)	7125.0 (1)	3.09 (1)
E = 1.41 V				
LiOH	n/a	n/a	n/a	n/a
NaOH	8344.5 (3)	2.75 (4)	7125.1 (1)	3.11 (1)
KOH	n/a	n/a	n/a	n/a
RbOH	8343.9 (1)	2.39 (8)	7125.3 (3)	3.13 (6)
CsOH	8344.2 (3)	2.55 (20)	7125.0 (1)	3.09 (1)
E = 1.48 V				
LiOH	8345.1 (1)	3.15 (6)	7124.9 (1)	3.06 (1)
NaOH	8346.0 (3)	3.69 (17)	7125.1 (1)	3.10 (1)
KOH	8345.8 (1)	3.52 (4)	7125.0 (1)	3.09 (1)
RbOH	8345.4 (1)	3.28 (9)	7125.2 (3)	3.12 (5)
CsOH	8345.8 (4)	3.52 (24)	7125.0 (1)	3.09 (1)
E = 1.56 V				
LiOH	8346.0 (1)	3.65 (7)	7124.9 (2)	3.06 (1)
NaOH	8346.2 (1)	3.84 (1)	7125.1 (1)	3.10 (1)
KOH	8345.9 (1)	3.64 (8)	7125.0 (1)	3.09 (1)
RbOH	8346.1 (1)	3.73 (8)	7125.2 (3)	3.12 (5)
CsOH	8346.2 (1)	3.82 (1)	7125.0 (1)	3.09 (1)
E = 1.66 V				
LiOH	8346.1 (3)	3.71 (6)	7124.9 (3)	3.07 (1)
NaOH	8346.3 (4)	3.82 (2)	7125.1 (1)	3.10 (1)
KOH	8346.2 (1)	3.75 (6)	7125.0 (1)	3.09 (1)
RbOH	8346.1 (2)	3.74 (13)	7125.2 (3)	3.13 (5)
CsOH	8346.3 (1)	3.85 (5)	7125.1 (1)	3.10 (1)
E = 1.1 V				
LiOH	8343.5 (2)	2.14 (10)	7124.8 (9)	3.07 (2)
NaOH	8343.7 (1)	2.28 (6)	7125.2 (1)	3.12 (2)
KOH	8343.7 (2)	2.26 (11)	7125.1 (1)	3.10 (1)
RbOH	8343.7 (1)	2.25 (4)	7125.3 (2)	3.13 (3)
CsOH	8343.9 (3)	2.35 (20)	7125.1 (1)	3.10 (1)

Edge positions were estimated at half-height of the edge jump.

[n/a] Not analyzed

Supplementary Table 4. Atomic coordination distances at the Ni *K*-edge of the $Ni_{65}Fe_{35}$ catalyst from simulations of the $(k)k^3$ weighted EXAFS using FEFF,[1] measured at the P64 beamline at Petra III (DESY). The measurements were carried out in purified 0.1 M alkali hydroxides. The oxidation states were obtained from the R_{Ni-O} coordination distances using calibration curves shown in Supplementary Fig. 14c. The selected k-range was 2-9 Å^{-1}, the Debye-Waller parameters were fixed to 0.071 Å for the Ni-O shell, and to 0.087 Å for the Ni-Ni shell, and the amplitude reduction factor (S_0^2) set to 0.85. The uncertainty of the last digit(s) is given in parenthesis, and shows the fit error (68 % confidence interval).

Ni *K*-edge									
	R_{Ni-O} /Å	N_{Ni-O}	R_{Ni-Ni} /Å	N_{Ni-Ni}	R_{Ni-O} /Å	N_{Ni-O}	R_f	ΔE_0	Ox. state (R_{Ni-O})
E = 1.1 V vs. RHE									
LiOH	2.039 (7)	6.0 (3)	3.057 (9)	4.7 (5)	3.59 (2)	7 (1)	19.5	0.7	2.11
NaOH	2.042 (7)	5.9 (3)	3.066 (9)	5.6 (5)	3.58 (3)	8 (2)	19.4	1.0	2.08
KOH	2.038 (7)	6.0 (3)	3.062 (9)	5.3 (5)	3.58 (2)	8 (2)	19.8	0.6	2.12
RbOH	2.033 (7)	5.9 (3)	3.060 (9)	5.0 (5)	3.56 (2)	7 (2)	19.4	0.3	2.18
CsOH	2.037 (6)	6.0 (2)	3.060 (8)	5.1 (5)	3.57 (2)	7 (1)	19.6	0.6	2.13
E = 1.34 V vs. RHE									
LiOH	2.040 (6)	6.0 (3)	3.055 (9)	4.7 (5)	3.59 (3)	7 (2)	19.7	0.7	2.10
NaOH	2.041 (7)	5.8 (3)	3.067 (9)	5.5 (5)	3.59 (3)	8 (2)	19.2	1.0	2.09
KOH	2.041 (7)	5.9 (3)	3.064 (9)	5.6 (5)	3.59 (3)	7 (2)	19.6	0.9	2.09
RbOH	2.036 (7)	5.9 (3)	3.056 (9)	5.3 (5)	3.57 (2)	7 (2)	19.5	0.8	2.14
CsOH	2.038 (6)	5.9 (2)	3.056 (8)	5.4 (5)	3.58 (2)	8 (1)	21.1	0.8	2.12
E = 1.41 V vs. RHE									
NaOH	1.971 (9)	6.0 *	2.968 (10)	6.0 *	3.51 (3)	4 (1)	16.4	-0.1	2.82
RbOH	2.023 (9)	5.0 (3)	3.053 (12)	3.9 (5)	3.57 (3)	7 (2)	21.9	-1.0	2.28
CsOH	2.004 (50)	6.0 *	2.904 (85)	6.0 *	3.57 (3)	6 *	14.8	0.7	2.48
E = 1.48 V vs. RHE									
LiOH	1.907 (8)	3.8 (2)	2.825 (10)	2.8 (3)	3.52 (2)	5 (1)	17.3	0.8	3.48
NaOH	1.890 (6)	4.7 (2)	2.830 (6)	6.1 (3)	3.50 (3)	5 (1)	10.8	2.6	3.65
KOH	1.893 (7)	4.4 (2)	2.823 (7)	5.0 (3)	3.51 (3)	4 (1)	11.5	1.7	3.62
RbOH	1.897 (8)	4.1 (2)	2.813 (8)	4.0 (3)	3.52 (3)	5 (1)	13.4	0.8	3.58
CsOH	1.893 (6)	4.4 (2)	2.812 (6)	5.0 (3)	3.53 (2)	5 (1)	16.1	1.2	2.62
E = 1.56 V vs. RHE									
LiOH	1.887 (6)	4.7 (2)	2.830 (6)	5.3 (3)	3.51 (3)	5 (1)	12.7	2.4	3.68
NaOH	1.886 (6)	5.0 (2)	2.828 (5)	6.7 (3)	3.49 (2)	6 (1)	11.9	2.8	3.69
KOH	1.887 (7)	4.6 (2)	2.821 (6)	5.5 (3)	3.49 (3)	4 (1)	11.7	1.9	2.68
RbOH	1.893 (6)	4.6 (2)	2.833 (6)	5.8 (3)	3.50 (3)	4 (1)	12.1	2.8	3.62
CsOH	1.887 (5)	5.0 (2)	2.820 (5)	6.7 (3)	3.51 (2)	5 (1)	13.8	2.2	3.68
E = 1.66 V vs. RHE									
LiOH	1.884 (6)	4.9 (2)	2.829 (6)	5.5 (3)	3.49 (2)	4 (1)	13.4	2.4	3.71
NaOH	1.885 (6)	5.0 (2)	2.829 (5)	6.8 (3)	3.49 (3)	6 (1)	12.2	2.9	3.70
KOH	1.885 (6)	4.9 (2)	2.824 (6)	6.1 (3)	3.49 (3)	4 (1)	12.0	2.3	3.70
RbOH	1.891 (6)	5.0 (2)	2.830 (5)	6.5 (3)	3.51 (2)	5 (1)	11.7	2.7	3.64
CsOH	1.884 (5)	5.1 (2)	2.818 (5)	6.6 (3)	3.50 (2)	4 (1)	13.6	2.1	3.71
E = 1.1 V vs. RHE (after OER)									
LiOH	2.036 (7)	6.1 (3)	3.053 (9)	4.4 (5)	3.57 (2)	8 (2)	20.0	0.6	2.14
NaOH	2.041 (7)	5.9 (3)	3.066 (9)	5.6 (5)	3.58 (2)	9 (2)	19.0	0.8	2.09
KOH	2.035 (7)	5.9 (3)	3.061 (9)	5.3 (5)	3.58 (2)	7 (2)	19.6	0.6	2.14
RbOH	2.038 (7)	6.0 (3)	3.059 (9)	5.7 (5)	3.58 (2)	7 (2)	19.4	0.7	2.12
CsOH	2.043 (6)	6.2 (2)	3.057 (8)	5.4 (5)	3.58 (2)	8 (1)	19.0	1.0	2.07

R = coordination distance, *N* = coordination number, σ = Debye-Waller parameter, ΔE_0 = energy correction factor

* The coordination number was fixed to 6 of some half-oxidized states that were too disordered.

Supplementary Table 5. Atomic coordination distances at the Fe *K*-edge of the $Ni_{65}Fe_{35}$ catalyst from simulations of the $(k)k^3$ weighted EXAFS using FEFF.[1] The measurements were carried out in purified 0.1 M of alkali hydroxides. The oxidation states were obtained from the $R_{Fe\text{-}O}$ coordination distances from the calibration curves in Supplementary Fig. 14d. The fitted k-range was 2-9 Å^{-1}, the Debye-Waller parameters were fixed to 0.092 Å for the Fe-O shell, and to 0.13 Å for the Fe-Ni shell, and the amplitude reduction factor (S_0^2) set to 0.85. Fit errors were estimated up to a distance of 4 Å. The data in this table is from the P64 beamline at Petra III (DESY). The uncertainty of the last digit(s) is given in parenthesis, and shows the fit error (68 % confidence interval).

Fe *K*-edge									
	$R_{Fe\text{-}O}$ /Å	$N_{Fe\text{-}O}$	$R_{Fe\text{-}Fe}$ /Å	$N_{Fe\text{-}Fe}$	$R_{Fe\text{-}O}$ /Å	$N_{Fe\text{-}O}$	R_f	ΔE_0	Ox. state ($R_{Fe\text{-}O}$)
E = 1.1 V vs. RHE									
LiOH	1.983 (10)	5.1 (3)	3.026 (17)	4 (1)	3.59 (7)	3 (2)	17.4	0.4	3.30
NaOH	1.992 (9)	5.5 (3)	3.057 (16)	5 (1)	3.60 (5)	4 (2)	15.1	0.6	3.20
KOH	1.990 (9)	5.3 (3)	3.044 (16)	5 (1)	3.59 (6)	3 (2)	16.1	0.6	3.22
RbOH	1.999 (9)	5.8 (3)	3.071 (13)	6 (1)	3.60 (4)	4 (2)	14.2	0.8	3.13
CsOH	1.987 (9)	5.2 (3)	3.037 (17)	5 (1)	3.60 (5)	3 (2)	17.4	0.4	3.25
E = 1.34 V vs. RHE									
LiOH	1.984 (10)	5.1 (3)	3.028 (16)	5 (1)	3.59 (7)	2 (2)	17.8	0.4	3.26
NaOH	1.991 (9)	5.7 (3)	3.062 (14)	6 (1)	3.61 (5)	4 (2)	14.5	0.4	3.21
KOH	1.992 (5)	5.3 (3)	3.046 (13)	5 (1)	3.59 (5)	3 (2)	16.0	0.6	3.20
RbOH	1.993 (9)	5.8 (3)	3.069 (13)	6 (1)	3.59 (4)	4 (2)	14.7	0.5	3.19
CsOH	1.987 (9)	5.2 (3)	3.042 (16)	5 (1)	3.59 (6)	3 (2)	16.7	0.4	3.25
E = 1.41 V vs. RHE									
NaOH	1.989 (9)	5.5 (3)	3.040 (17)	4 (1)	3.58 (4)	4 (2)	15.0	0.5	3.23
RbOH	1.995 (5)	5.7 (3)	3.065 (12)	6 (1)	3.59 (3)	4 (2)	15.7	0.6	3.19
CsOH	1.984 (10)	5.1 (3)	3.028 (17)	4 (1)	3.58 (5)	3 (2)	18.1	0.3	2.39
E = 1.48 V vs. RHE									
LiOH	1.971 (0.010)	4.9 (3)	2.988 (18)	4 (1)	3.58 (6)	3 (2)	19.4	-0.1	3.42
NaOH	1.949 (9)	4.9 (3)	2.900 (13)	5 (1)	3.57 (4)	4 (1)	15.9	-0.4	3.66
KOH	1.962 (5)	4.9 (3)	2.955 (17)	4 (1)	3.56 (4)	4 (2)	18.8	-0.3	3.52
RbOH	1.945 (8)	5.1 (3)	2.889 (12)	5 (1)	3.57 (3)	5 (1)	14.4	-0.5	3.87
CsOH	1.947 (9)	4.8 (3)	2.915 (19)	4 (1)	3.55 (4)	4 (1)	18.8	-0.9	3.68
E = 1.56 V vs. RHE									
LiOH	1.961 (10)	4.7 (3)	2.946 (19)	4 (1)	3.55 (5)	3 (1)	19.6	-0.2	3.53
NaOH	1.942 (9)	4.8 (3)	2.895 (13)	5 (1)	3.55 (4)	4 (1)	15.9	-0.6	3.73
KOH	1.948 (10)	4.7 (3)	2.918 (17)	4 (1)	3.55 (4)	4 (1)	19.3	-0.7	3.67
RbOH	1.929 (8)	5.1 (3)	2.867 (10)	7 (1)	3.55 (3)	5 (1)	12.0	-0.8	3.89
CsOH	1.940 (9)	4.7 (3)	2.901 (15)	4 (1)	3.55 (4)	4 (1)	17.5	-0.9	3.76
E = 1.66 V vs. RHE									
LiOH	1.958 (10)	4.7 (3)	2.951 (18)	4 (1)	3.55 (5)	3 (2)	19.8	-0.3	3.56
NaOH	1.936 (10)	4.7 (3)	2.889 (13)	6 (1)	3.55 (5)	3 (2)	14.7	-4.8	3.80
KOH	1.950 (10)	4.7 (3)	2.917 (18)	4 (1)	3.54 (4)	4 (1)	18.7	-0.5	3.65
RbOH	1.927 (9)	5.0 (3)	2.868 (10)	8 (1)	3.56 (3)	5 (2)	11.8	-4.6	3.89
CsOH	1.937 (10)	4.7 (3)	2.895 (16)	5 (1)	3.54 (4)	4 (2)	17.5	-4.9	3.79
E = 1.1 V vs. RHE (after OER)									
LiOH	1.977 (10)	4.8 (3)	3.002 (22)	3 (1)	3.58 (4)	4 (2)	18.4	0.2	3.36
NaOH	1.987 (9)	5.5 (3)	3.052 (15)	5 (1)	3.60 (5)	3 (2)	16.1	0.2	3.25
KOH	1.999 (9)	5.3 (3)	3.044 (15)	5 (1)	3.60 (6)	3 (2)	15.9	0.6	3.22
RbOH	2.001 (8)	6.0 (3)	3.072 (12)	7 (1)	3.60 (5)	3 (2)	13.5	0.9	3.10
CsOH	1.987 (9)	5.3 (3)	3.037 (15)	5 (1)	3.60 (6)	3 (2)	17.0	0.4	3.25

R = coordination distance, *N* = coordination number, σ = Debye-Waller parameter, ΔE_0 = energy correction factor
* The coordination number was fixed to 6 for the half-oxidized states, which were too disordered.

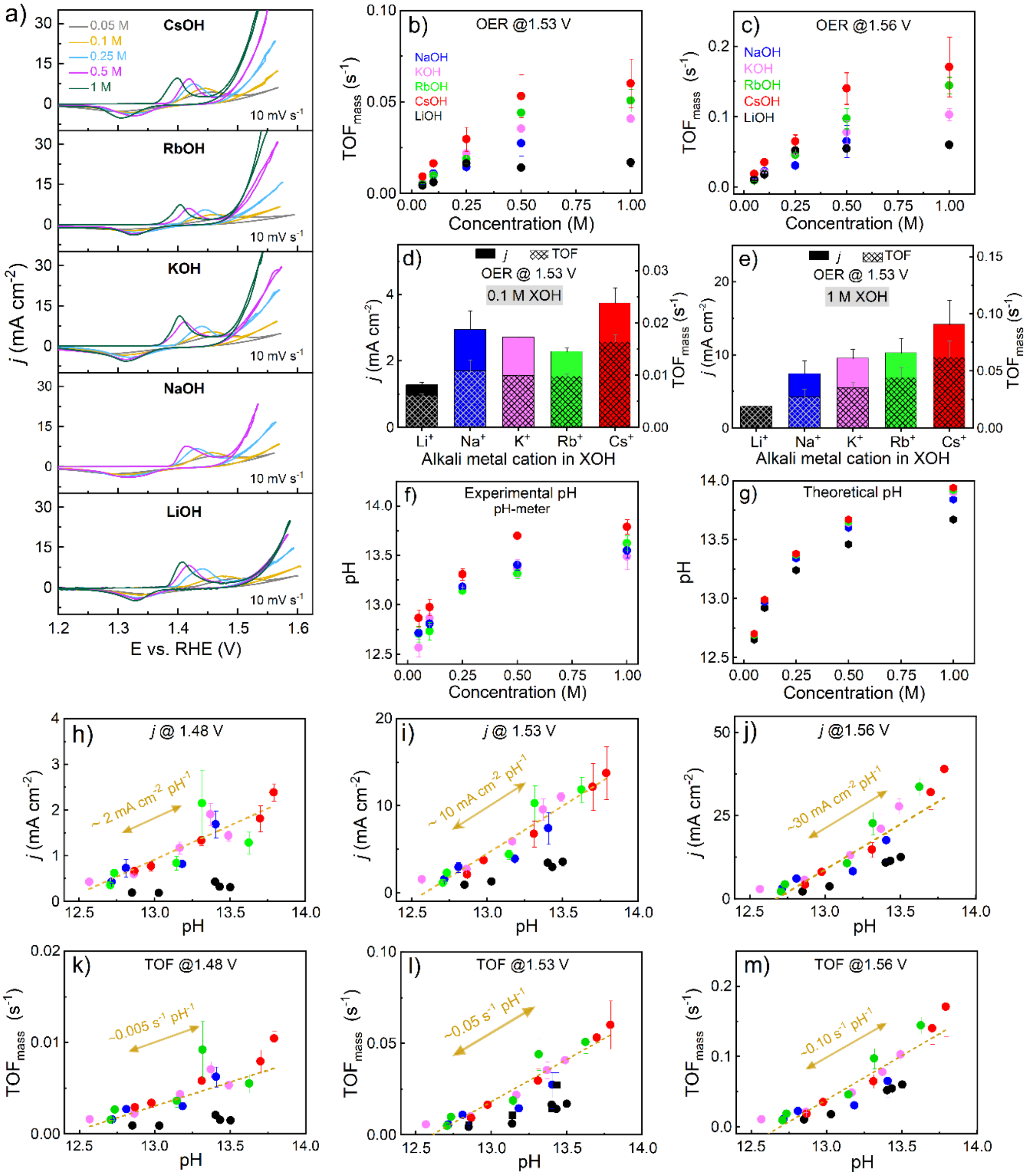

Supplementary Fig. 15. The influence of alkali hydroxide concentration on the OER activity. Measurements of the of the $Ni_{65}Fe_{35}$ catalyst in purified 0.05 M, 0.1 M, 0.25 M, 0.5 M and 1 M hydroxides (LiOH, RbOH, KOH, NaOH, and CsOH). **(a)** CVs at 10 mV s^{-1}. **(b)** TOF_{mass} at 1.53 V vs. the concentration. **(c)** TOF_{mass} at 1.56 V vs. the concentration. **(d)** Current density (j) and TOF_{mass} at 1.53 V in 0.1 M hydroxides. **(e)** j and TOF_{mass} at 1.53 V in 1 M hydroxides. **(f)** Experimental pH of the alkali hydroxides measured with a pH-meter (LiOH is excluded from this plot due to erroneous reading but the values are shown in Supplementary Table 6). **(g)** Theoretical pH of the alkali hydroxides calculated from their pK_b values using Supplementary Equations (1)-(13). **(h)** j at 1.48 V vs. the electrolyte pH. **(i)** j at 1.53 V vs. the pH. **(j)** j at 1.56 V vs. the pH. **(k)** TOF_{mass} at 1.48 V vs. the pH. **(l)** TOF_{mass} at 1.53 V vs. the pH. **(m)** TOF_{mass} at 1.56 V vs. the pH. The pH of LiOH in figures (h)-(m) was determined from the calibration curve in Supplementary Fig. 16, and represents an approximate value. Error bars represent the standard error from the overall number of measurements.

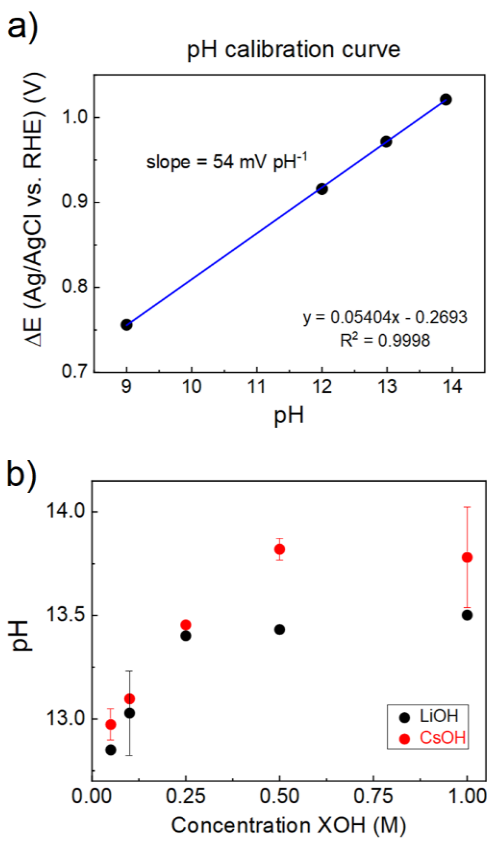

Supplementary Fig. 16. pH calibration curve **(a)** The potential difference between the reversible hydroxgen electrode (RHE) and the leak-free Ag/AgCl reference electrode, obtained using commercial buffer solution pH 9 (Certipur®, traceable to SRM from NIST and PTB, Merck), buffer solution pH 12 (Reagecon, traceable to NIST), and commercial KOH solution 0.1 M (pH 12.99) and 1 M (pH 13.90) (Titripur®, Supelco). The pH of the commercial KOH was assumed as the theoretical values according to Supplementary Equations (1)-(13). **(b)** The obtained pH values of LiOH and CsOH using the calibration curve presented in (a). Error bars represent the standard error from the overall number of measurements.

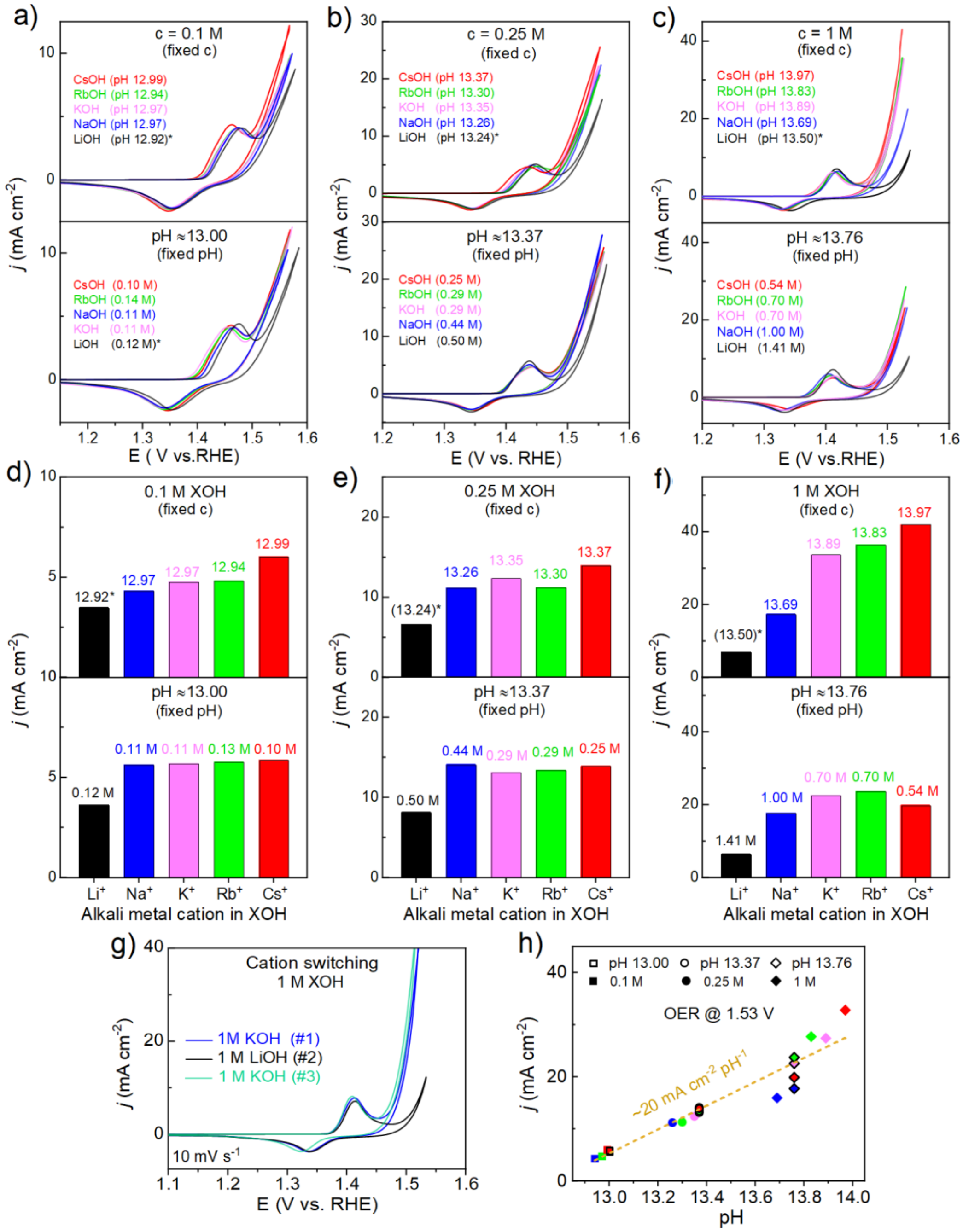

Supplementary Fig. 17. OER activity at fixed pH or concentration in freshly prepared (non-purified) alkali hydroxides. **(a)** CVs at 10 mV s^{-1} in 0.1 M (top) or at a fixed pH of ~13.00 (bottom) **(b)** CVs at 10 mV s^{-1} in 0.25 M (top) or at a fixed pH of ~13.37 (bottom) **(c)** CVs at 10 mV s^{-1} in 1 M (top) or at a fixed pH of ~13.76 (bottom). **(d)** OER activities at 1.53 V extracted from the CVs in (a) for a brief comparison: in 0.1 M (top) or at pH ~13.00 (bottom) **(e)** the corresponding OER activities in 0.25 M (top) or at ~pH 13.37 (bottom) **(f)** and the OER activities in 1 M (top) or at pH ~13.76 (bottom). **(g)** A cross-check of the “cation switching” protocol used in (a)-(c) in the order of KOH – LiOH – KOH. **(h)** OER activity at 1.53 V vs. the electrolyte pH (the values in LiOH are omitted for clarity). All measurements in this figure were carried out by a “cation switching” protocol to avoid discrepancies in the film thickness and thus in activity. The same film was used for different cations to avoid differences in loading; however, a new film was used for each pH-range. The films were rinsed carefully with Milli-Q water between switching. A special pH-electrode was used for these measurements, which had a ceramic membrane designed to minimize the alkali error (InLab® Routine Pro-ISM, Mettler-Toledo). Despite this, we encountered the same problem in LiOH, and the pH could not be determined. *The reported pH in LiOH is therefore the theoretical value.

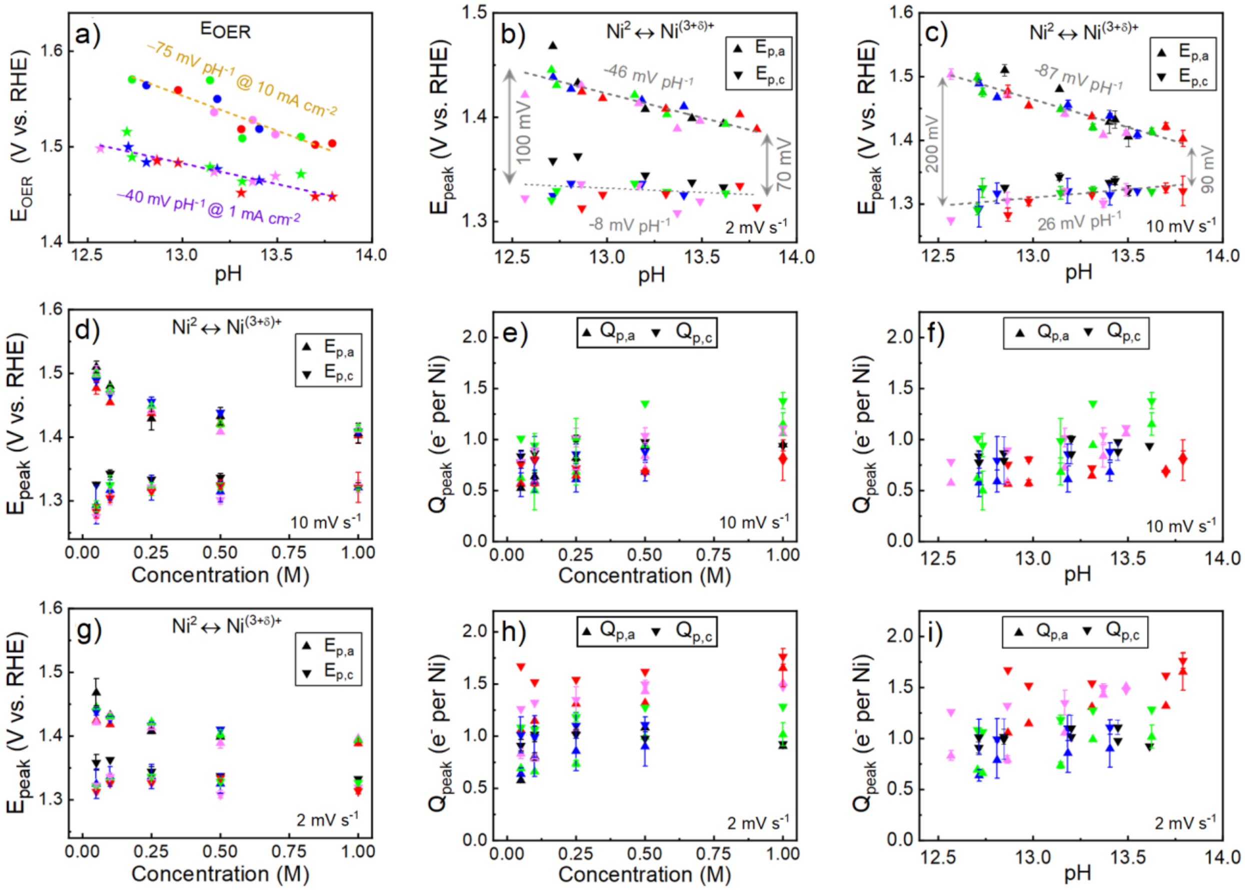

Supplementary Fig. 18. Influence of the electrolyte pH on the OER activity and peak potential of the $Ni_{65}Fe_{35}$ catalyst. **(a)** The OER overpotential extracted at two different current densities (1 mA cm^{-2} and 10 mA cm^{-2}), plotted vs. the pH. **(b)** Anodic ($E_{p,a}$) and cathodic ($E_{p,c}$) redox peak positions of the $Ni^{2+/(3+\delta)+}$ couple determined from CVs at 2 mV s^{-1} shown vs. the experimental pH. **(c)** Peak potential from CVs at 10 mV s^{-1} vs. the experimental pH. **(d)** Peak potential from CVs at 10 mV s^{-1} vs. the concentration. **(e)** The integrated redox peak area (charge, Q_{peak}) from CVs at 10 mV s^{-1} in units ´moles of electrons transferred per moles of Ni ions´ (e$^-$ per Ni), plotted vs. the concentration. **(f)** Q_{peak} from CVs at 10 mV s^{-1} vs. the electrolyte pH. **(g)** Peak potential from CVs at 2 mV s^{-1} vs. the concentration. **(h)** Q_{peak} from CVs at 2 mV s^{-1} vs. the concentration. **(i)** Q_{peak} from CVs at 2 mV s^{-1} vs. the electrolyte pH. Note that the pH of LiOH is determined from the offset between the RHE and the Ag/AgCl electrodes, whereas the pH of the other alkali hydroxides was determined using a pH-meter. Error bars represent the standard error from the overall number of measurements.

Supplementary Table 6. Concentrations and electrolyte pH for the alkali metal hydroxides at different molar concentrations. Theoretical pH and dissociated [OH^-] determined from Supplementary Equations (1)-(13) and the tabulated pK_b values. The experimental pH determined using a standard pH-meter, and the pH determined using the offset of the RHE electrode against a reversible Ag/AgCl reference electrode. The uncertainty of the last digit(s) is given in parenthesis, and shows the standard error.

	LiOH	NaOH	KOH	RbOH	CsOH
Cation radius (pm) [(a)]	69	102	138	149	170
pK_a [(b)]	13.6	14.2	14.5	14.6	14.8
pK_b [(c)]	0.4	-0.2	-0.5	-0.6	-0.8
**c (M)**	**Experimental pH (pH-meter)**				
0.05	11.97 (2) [(d)]	12.71 (2)	12.57 (9)	12.70 (6)	12.87 (6)
0.1	11.83 (6) [(d)]	12.88 (6)	12.86 (6)	12.77 (7)	12.98 (8)
0.25	11.38 (5) [(d)]	13.16 (2)	13.17 (1)	13.14 (7)	13.31 (7)
0.5	11.01 (3) [(d)]	13.38 (4)	13.37 (9)	13.32 (4)	13.70 (3)
1	10.85 (14) [(d)]	13.55 (7)	13.50 (13)	13.63 (7)	13.79 (7)
**c (M)**	**Theoretical pH from equations (1)-(13)**				
0.05	12.65	12.69	12.69	12.69	12.70
0.1	12.92	12.97	12.99	12.99	12.99
0.25	13.24	13.34	13.37	13.37	13.38
0.5	13.46	13.60	13.64	13.65	13.67
1	13.67	13.84	13.90	13.92	13.94
**c (M)**	**Theoretical dissociated [OH^-] (M)**				
0.05	0.045	0.049	0.049	0.049	0.050
0.1	0.083	0.094	0.097	0.098	0.098
0.25	0.17	0.220	0.23	0.24	0.24
0.5	0.29	0.40	0.44	0.45	0.47
1	0.46	0.70	0.80	0.83	0.88
**c (M)**	**Experimental pH (offset of RHE vs. Ag/AgCl)**				
0.05	12.85	n/a [(e)]	n/a [(e)]	n/a [(e)]	12.97
0.1	13.03	n/a [(e)]	n/a [(e)]	n/a [(e)]	13.10
0.25	13.40	n/a [(e)]	n/a [(e)]	n/a [(e)]	13.45
0.5	13.43	n/a [(e)]	n/a [(e)]	n/a [(e)]	13.82
1	13.50	n/a [(e)]	n/a [(e)]	n/a [(e)]	13.78

[n/a)] No value, se individual label for more information.

[(a)] The cation radius was obtained from Marcus et al.[8]

[(b)] The pK_a values were obtained from Baes et al. [13]

[(c)] The pK_b values were calculated from the pK_a values (pK_w = pK_a + pK_b), where pK_w = 14

[(d)] Erroneous reading in LiOH with a pH-meter due to the small size of the Li^+ ion

[(e)] Not analyzed

Supplementary Table 7. The concentration of cations (Li^+, Na^+, K^+, and Rb^+) in the alkali hydroxide electrolytes determined using ICP-OES. The concentration of for Cs^+ could not be determined due to low emission intensity. The electrolyte pH was calculated using the Supplementary Equations (1)-(13). Note that alkali metal cations are easily ionized elements (EIS), so a certain error needs to be considered (not estimated). Also, every cation has a different dilution factor since ICP is measured in wt % (related to the differences in the molecular weight between the hydroxides), which introduce further error (see more information in the Methods section).

	**Li**	**Na**	**K**	**Rb**	**Cs**	**Ni**	**Fe**
c (M)	Cation concentration from ICP-OES (M)						
0.1 (*purified)	0.061	0.094	0.085	0.079	n/a [(a)]	n/a [(b)]	n/a [(b)]
1 (*purified)	0.61	0.91	0.85	0.80	n/a [(a)]	n/a [(b)]	n/a [(b)]
c (M)	Calculated pH						
0.1 (*purified)	12.70	12.95	12.91	12.89	n/a [(a)]	n/a [(b)]	n/a [(b)]
1 (purified)	13.45	13.80	13.83	13.82	n/a [(a)]	n/a [(b)]	n/a [(b)]

n/a) no value obtained or not analyzed, se label for more information.

a) The Cs^+ ion has close to zero emission intensity and could not be detected.

b) The values were below the detection limit.

Supplementary Table 8. OER activity parameters in different concentrations of the purified alkali hydroxides. The data reported in this table was measured "in house" using an RHE reference electrode. All activity data is obtained from steady-state conditions. The peak positions were obtained from CVs at 10 mV s^{-1}. The R_u values (iR-drop) were determined using electrochemical impedance spectroscopy (EIS). The uncertainty of the last digit(s) is given in parenthesis, and shows the standard error. The turnover frequency (TOF_{mass}) is calculated based on the total metal loading (Ni and Fe content) obtained from ICP-OES.

XOH	c (M)	R_u (Ω)	*j* @ 1.48 V (mA cm^{-2})	*j* @ 1.53 V (mA cm^{-2})	*j* @ 1.56 V (mA cm^{-2})	TOF @ 1.48 V (s^{-1})	TOF @ 1.53 V (s^{-1})	TOF @ 1.56 V (s^{-1})	$E_{p,a}$ (V vs. RHE)	$E_{p,c}$ (V vs. RHE)
LiOH	0.05 M	298 (6)	0.19 (1)	0.90 (3)	2.2 (1)	0.0009 (1)	0.0043 (2)	0.010 (1)	1.510 (6)	1.326 (1)
NaOH	0.05 M	261 (12)	0.43 (2)	1.52 (15)	3.0 (4)	0.0016 (1)	0.0056 (6)	0.011 (2)	1.489 (4)	1.293 (30)
KOH	0.05 M	308 (6)	0.43 (2)	1.53 (1)	2.9 (3)	0.0016 (1)	0.0057 (1)	0.01 (1)	1.503 (8)	1.275 (2)
RbOH	0.05 M	320 (10)	0.36 (7)	1.14 (14)	2.2 (2)	0.0015 (3)	0.0049 (6)	0.009 (1)	1.499 (6)	1.291 (9)
CsOH	0.05 M	196 (8)	0.66 (7)	2.10 (32)	4.2 (6)	0.0029 (3)	0.0092 (14)	0.019 (3)	1.477(10)	1.283 (10)
LiOH	0.1 M	138 (5)	0.19 (4)	1.3 (1)	3.7 (2)	0.0009 (3)	0.0061 (4)	0.018 (1)	1.480 (1)	1.434 (5)
NaOH	0.1 M	147 (8)	0.73 (9)	3.0 (6)	6.1 (8)	0.0027 (7)	0.011 (2)	0.022 (3)	1.467 (3)	1.317 (16)
KOH	0.1 M	145 (2)	0.60 (2)	2.7 (1)	5.7 (1)	0.0022 (1)	0.010 (1)	0. 021 (1)	1.471 (1)	1.306 (13)
RbOH	0.1 M	178 (6)	0.62 (2)	2.3 (1)	4.3 (1)	0.0027 (1)	0.0098 (5)	0.019 (1)	1.475 (7)	1.325 (15)
CsOH	0.1 M	106 (3)	0.77 (10)	3.8 (3)	8.1 (6)	0.0034 (4)	0.016 (7)	0.035(10)	1.454 (1)	1.304 (8)
LiOH	0.25 M	67 (1)	0.42 (5)	3.3 (3)	9 (1)	0.0021 (3)	0.016 (1)	0.039 (2)	1.429 (13)	1.334 (1)
NaOH	0.25 M	68 (4)	0.82 (1)	3.9 (1)	8 (1)	0.0030 (1)	0.014 (1)	0.031 (1)	1.456 (7)	1.320 (20)
KOH	0.25 M	67 (3)	1.2 (1)	5.9 (2)	13 (1)	0.0043 (1)	0.022 (1)	0.049 (1)	1.442 (5)	1.321 (3)
RbOH	0.25 M	80 (3)	0.8 (2)	4.4 (5)	11 (1)	0.0036 (7)	0.019 (2)	0.046 (4)	1.449 (5)	1.318 (10)
CsOH	0.25 M	57 (1)	1.3 (1)	6.8 (1)	15 (1)	0.0058 (5)	0.030 (7)	0.065 (10)	1.437 (2)	1.315 (2)
LiOH	0.5 M	40 (1)	0.32 (3)	3 (1)	11 (1)	0.0015 (1)	0.014 (1)	0.055 (3)	1.433 (9)	1.337 (6)
NaOH	0.5 M	45 (5)	1.7 (3)	7 (2)	18 (6)	0.0062 (1)	0.027 (7)	0.065 (23)	1.439 (3)	1.315 (17)
KOH	0.5 M	50 (7)	1.9 (2)	10 (1)	21 (4)	0.0071 (1)	0.035 (5)	0.078 (16)	1.408 (1)	1.302 (7)
RbOH	0.5 M	41 (2)	2.1 (7)	10 (2)	23 (3)	0.0092 (31)	0.044 (9)	0.097 (14)	1.420 (5)	1.322 (6)
CsOH	0.5 M	30 (1)	1.8 (3)	12 (3)	32 (5)	0.0079(13)	0.053 (12)	0.14 (2)	1.422 (5)	1.325 (7)
LiOH	1 M	27 (1)	0.31 (1)	4 (1)	13 (1)	0.0015 (1)	0.017 (2)	0.060 (4)	1.406 (11)	1.320 (8)
NaOH	1 M	n/a	n/a	n/a	n/a	n/a	n/a	n/a	1.409 (6)	1.321 (1)
KOH	1 M	22 (1)	1.4 (1)	11(1)	28 (2)	0.0053 (4)	0.041 (2)	0.10 (1)	1.411 (4)	1.322 (10)
RbOH	1 M	21 (1)	1.3 (2)	12 (1)	34 (3)	0.006 (1)	0.051 (6)	0.14 (1)	1.414 (5)	1.320 (2)
CsOH	1 M	21 (1)	2.4 (2)	14 (3)	39 (9)	0.010 (1)	0.060 (13)	0.17 (4)	1.403(12)	1.321 (23)

n/a) not measured

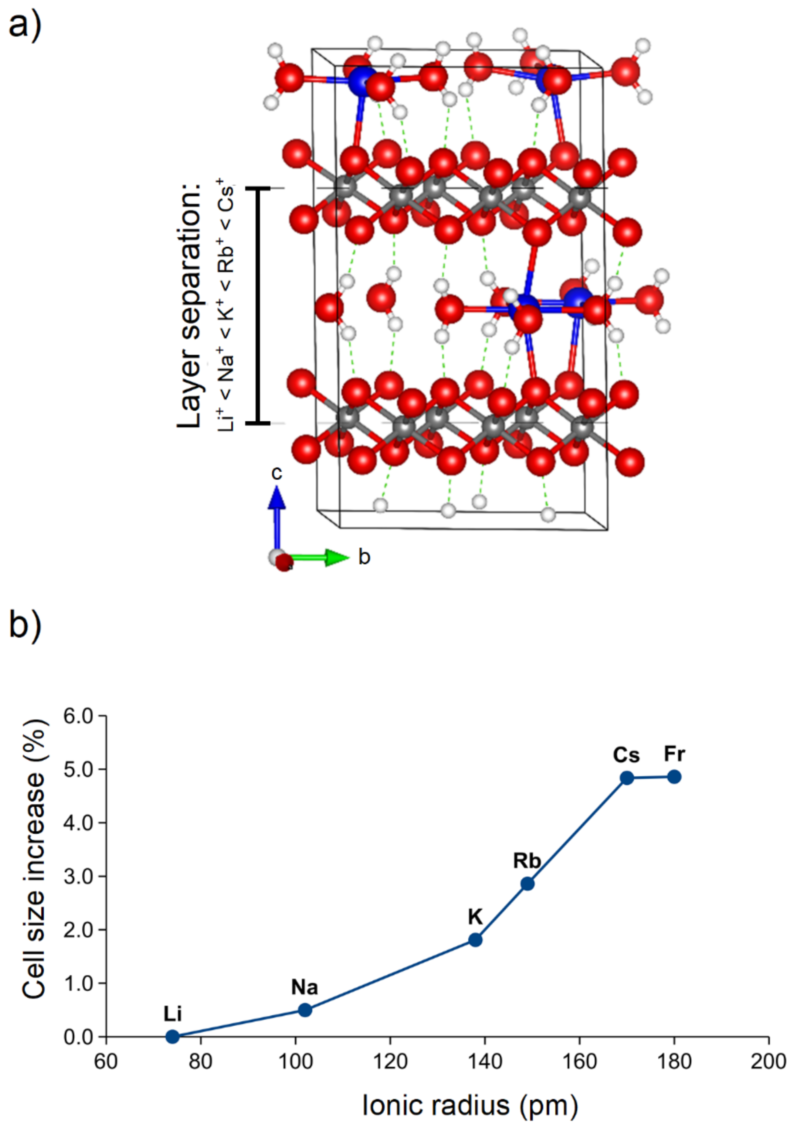

Supplementary Fig. 19. Cation NiOOH cell size. **(a)** Representations of the γ-NiOOH bulk structure. The effect of the intercalating cation on the layer spacing is indicated, with Li and Cs resulting in the smallest and largest separation, respectively. The atoms are shown using the colour code; Ni=grey, O=red, Alkali=blue, H=white). **(b)** Difference (%) wrt to Li versus ionic radius estimated using DFT calculations as a function of the intercalating alkali metal cation.

Supplementary Table 9. DFT-derived cation (X)-oxygen (O) coordination distances to either the surrounding water molecules (denoted "X-OH_2") or to the lattice oxygens of the surface of the Ni(Fe)OOH (1 0 0) (denoted "X-O"). The reported distances are the average values of all distances to the nearest oxygens.

X-O coordination distance	Li^+	Na^+	K^+	Rb^+	Cs^+
X-OH_2 (Å) (water)	2.35	2.36	2.39	2.42	2.46
X-O (Å) (lattice)	2.60	2.61	2.64	2.67	2.72

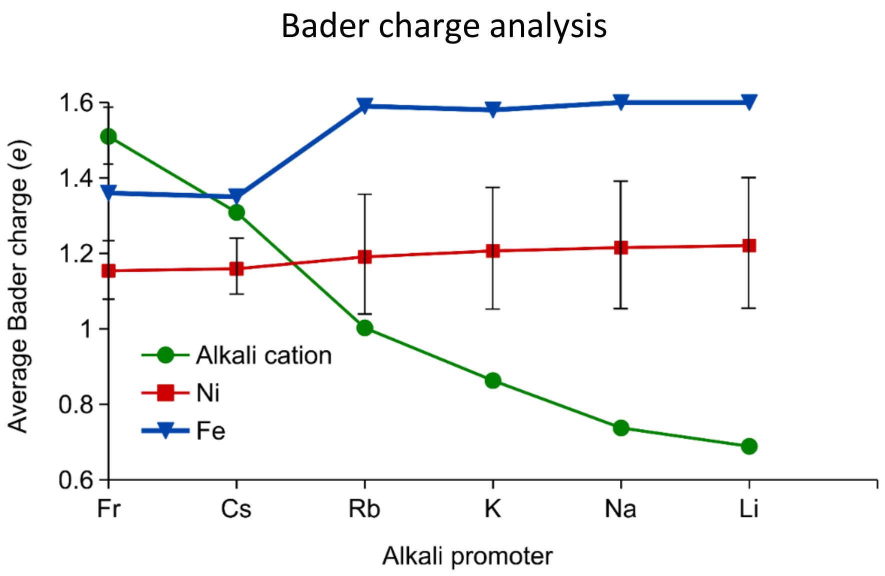

Supplementary Fig. 20. Average Bader charge analysis for the Ni- and Fe-sites in the bulk structure of water/alkali ion intercalated NiOOH and Fe-substituted Ni(Fe)OOH. The partial charge increases on the alkali cations from Li^+ to Cs^+ (green). For both Ni sites (red) and Fe sites (blue), the average partial charges remain largely unaffected. The error bars show the standard error (the variation) of the ions.

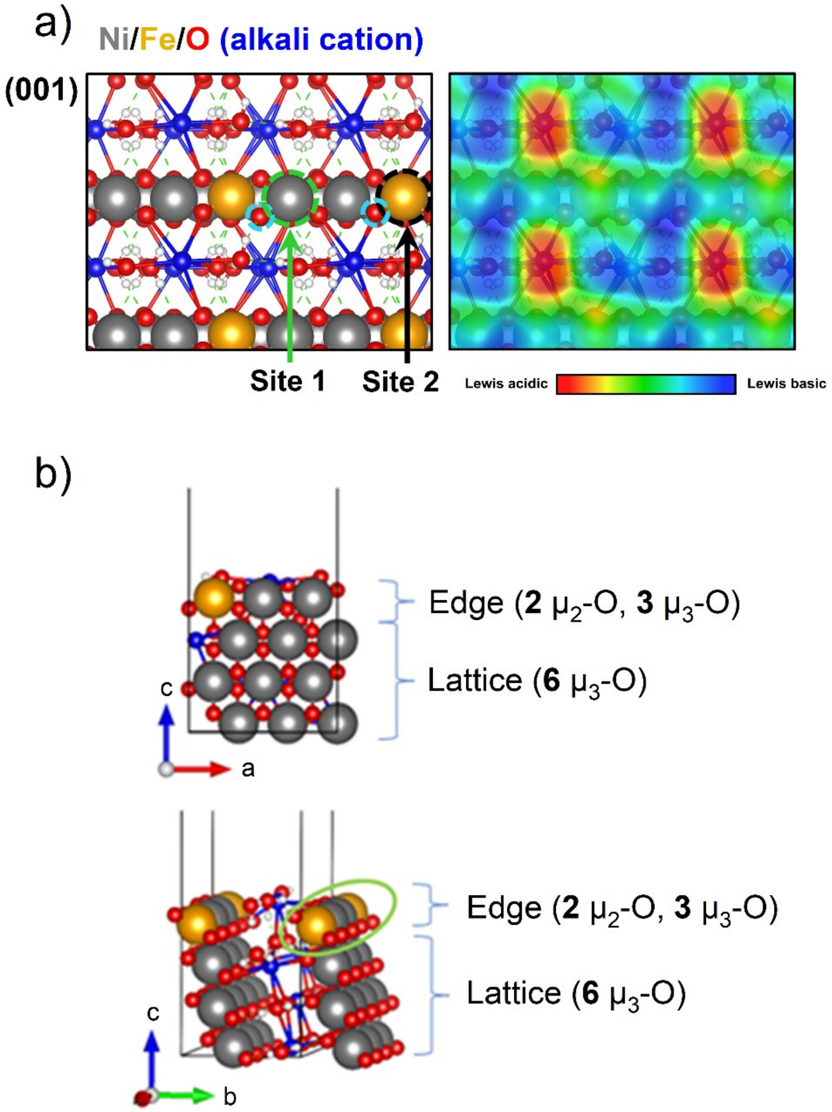

Supplementary Fig. 21. Surface model of Fe-doped Ni(Fe)OOH(100) **(a)** Model showing the Ni/Fe/O sites (and alkali cation) analyzed by the reactivity property approach marked (left). The atoms are shown using the colour code; Ni=grey, Fe=orange, O=red, Alkali=blue, H=white). The top-view of the surface with reactivity properties estimated using DFT mapped on the 0.001 au isodensity contour plot (right). **(b)** The coordination geometry for Site 1 and Site 2 in the model are indicated with arrows. The main difference between site 1 and 2 is the coordination environment of H_2O and the alkali metal cation. Both the Site 1 and 2 are "edge sites" with 2 μ_2-O and 3 μ_3-O oxygen sites, and all the other lattice sites in the model have 6 coordinatively saturated μ_3-O sites. Note that due to the positions of the alkali cations and the local water coordination, there is a significant difference between site 1 and 2, which is why we have decided to analyze them separately.

Supplementary Table 10. Local reactivity parameters for pure NiOOH given in electronvolt (eV). See Supplementary Fig. 21 vide supra for reference to sites. The listed values are the charge transfer capacity of the metal site ($E_{S,min}$), the maximum value reflecting the surface electrostatic potential ($V_{S,max}$) which measures the electrostatic contribution to the local Lewis acidity at the metal site, and the minima in the surface electrostatic potential of the oxygen site ($V_{S,min}$) and the surface local average ionization energy ($\bar{I}_{S,min}$) which indicate the electrostatic and charge-transfer contribution to the Lewis basicity of the oxygen site. The arrows given in brackets indicate the direction of change; lower value (↓), and higher value (↑).

Descriptor	Descriptor property	Li^+	Na^+	K^+	Rb^+	Cs^+
Site 1		Ni	Ni	Ni	Ni	Ni
$E_{S,min}$	More Lewis acidic (↓) M-site	-3.05	-3.01	-3.38	*-3.68*	*-4.26*
$V_{S,max}$	More Lewis acidic (↑) M-site	0.58	0.56	0.57	0.61	0.67
$V_{S,min}$	More Lewis basic (↓) O-site	-1.00	-1.01	-1.02	-1.01	-0.99
$\bar{I}_{S,min}$	More Lewis basic (↓) O-site	7.66	7.62	7.56	7.54	7.56
Site 2		Ni	Ni	Ni	Ni	Ni
$E_{S,min}$	More Lewis acidic (↓) M-site	-4.56	-4.44	-4.72	*-5.03*	*-5.66*
$V_{S,max}$	More Lewis acidic (↑) M-site	0.83	0.81	0.83	0.86	0.92
$V_{S,min}$	More Lewis basic (↓) O-site	-0.37	-0.38	-0.38	-0.38	-0.38
$\bar{I}_{S,min}$	More Lewis basic (↓) O-site	7.63	7.58	7.53	7.51	7.54

Supplementary Table 11. Local reactivity parameters for Fe-doped Ni(Fe)OOH given in electronvolt (eV). See Supplementary Fig. 21 vide supra for reference to sites. Site 1 was exchanged for an Fe-atom, and all activity descriptors re-investigated for the Ni-site next to the Fe-site. Then, site 2 was exchanged for an Fe atom and all activity descriptors for the Fe-site next to an Ni-site were determined. The effect Fe exchange in either Site 1 or 2 is directly compared for the K^+ electrolyte; showing that the Fe-site is always the most Lewis acidic site. The listed values are the charge transfer capacity of the metal site ($E_{S,min}$), the maximum value reflecting the surface electrostatic potential ($V_{S,max}$) which measures the electrostatic contribution to the local Lewis acidity at the metal site, and the minima in the surface electrostatic potential of the oxygen site ($V_{S,min}$) and the surface local average ionization energy ($\bar{I}_{S,min}$) which indicate the electrostatic and charge-transfer contribution to the Lewis basicity of the oxygen site. The arrows given in brackets indicate the direction of change; lower value (↓), and higher value (↑).

Descriptor	Property	Li^+	Na^+	K^+ (Fe at site1)	K^+ (Fe at site2)	Rb^+	Cs^+
**Site 1**		Ni	Ni	Fe	Ni	Ni	Ni
$E_{S,min}$	More Lewis acidic (↓) M-site	-3.82	-3.86	-10.63	-4.28	-4.56	-5.25
$V_{S,max}$	More Lewis acidic (↑) M-site	0.75	0.76	1.66	0.78	0.81	0.86
$V_{S,min}$	More Lewis basic (↓) O-site	-0.95	-0.95	-0.77	-0.94	-0.94	-0.93
$\bar{I}_{S,min}$	More Lewis basic (↓) O-site	7.65	7.63	7.79	7.58	7.58	7.56
**Site 2**		Fe	Fe	Ni	Fe	Fe	Fe
$E_{S,min}$	More Lewis acidic (↓) M-site	-12.29	-12.28	-5.70	-12.89	-13.24	-14.13
$V_{S,max}$	More Lewis acidic (↑) M-site	1.88	1.89	0.98	1.92	1.94	2.03
$V_{S,min}$	More Lewis basic (↓) O-site	-0.13	-0.12	-0.31	-0.11	-0.13	-0.14
$\bar{I}_{S,min}$	More Lewis basic (↓) O-site	7.88	7.85	7.56	7.79	7.80	7.78

Supplementary Table 12. Local reactivity parameters for pure NiOOH given in electronvolt (eV) on structures optimised including D3(BJ) dispersion corrections.[14,15] See Supplementary Fig. 21 vide supra for reference to sites. The listed values are the charge transfer capacity of the metal site ($E_{S,min}$), the maximum value reflecting the surface electrostatic potential ($V_{S,max}$) which measures the electrostatic contribution to the local Lewis acidity at the metal site, and the minima in the surface electrostatic potential of the oxygen site ($V_{\mathrm{S,min}}$) and the surface local average ionization energy ($\bar{I}_{\mathrm{S,min}}$) which indicate the electrostatic and charge-transfer contribution to the Lewis basicity of the oxygen site. The arrows given in brackets indicate the direction of change; lower value (↓), and higher value (↑).

Descriptor	Property	Li^+	Na^+	K^+	Rb^+	Cs^+
Site 1		Ni	Ni	Ni	Ni	Ni
$E_{S,min}$	More Lewis acidic (↓) M-site	-3.00	-2.99	-3.35	-3.62	-4.08
$V_{S,max}$	More Lewis acidic (↑) M-site	0.57	0.55	0.57	0.60	0.66
$V_{S,min}$	More Lewis basic (↓) O-site	-0.98	-1.00	-1.00	-0.99	-0.99
$\bar{I}_{S,min}$	More Lewis basic (↓) O-site	7.65	7.57	7.57	7.54	7.56
Site 2		Ni	Ni	Ni	Ni	Ni
$E_{S,min}$	More Lewis acidic (↓) M-site	-4.52	-4.35	-4.70	-4.95	-5.45
$V_{S,max}$	More Lewis acidic (↑) M-site	0.83	0.81	0.82	0.85	0.90
$V_{S,min}$	More Lewis basic (↓) O-site	-0.37	-0.37	-0.37	-0.37	-0.37
$\bar{I}_{S,min}$	More Lewis basic (↓) O-site	7.63	7.58	7.53	7.52	7.51

Supplementary Table 13. Structural difference (in vol-%) of the pure NiOOH bulk structure with intercalating cations for structures optimized with D3(BJ) dispersion corrections compared to without corrections.[14,15]

Li^+	Na^+	K^+	Rb^+	Cs^+
99.996	99.800	99.861	99.785	99.354

Supplementary Table 14. Local reactivity parameters for NiOOH given in electronvolt (eV) including implicit solvation effects vis the VASPSOL method.[16] See Supplementary Fig. 21 vide supra for reference to sites. The listed values are the charge transfer capacity of the metal site ($E_{S,min}$), the maximum value reflecting the surface electrostatic potential ($V_{S,max}$) which measures the electrostatic contribution to the local Lewis acidity at the metal site, and the minima in the surface electrostatic potential of the oxygen site ($V_{S,\mathrm{min}}$) and the surface local average ionization energy ($\bar{I}_{S,\mathrm{min}}$) which indicate the electrostatic and charge-transfer contribution to the Lewis basicity of the oxygen site. The arrows given in brackets indicate the direction of change; lower value (↓), and higher value (↑).

Descriptor	Property	**Li^+**	**Na^+**	**K^+**	**Rb^+**	**Cs^+**
Site 1		Ni	Ni	Ni	Ni	Ni
$E_{S,min}$	More Lewis acidic (↓) M-site	-3.08	-2.98	-2.99	-3.15	-3.51
$V_{S,max}$	More Lewis acidic (↑) M-site	0.50	0.52	0.55	0.65	0.75
$V_{S,min}$	More Lewis basic (↓) O-site	-1.26	-1.28	-1.27	-1.27	-1.29
$\bar{I}_{S,min}$	More Lewis basic (↓) O-site	7.59	7.53	7.43	7.38	7.42
Site 2		Ni	Ni	Ni	Ni	Ni
$E_{S,min}$	More Lewis acidic (↓) M-site	-4.26	-4.22	-4.38	-4.63	-5.02
$V_{S,max}$	More Lewis acidic (↑) M-site	1.03	1.09	1.14	1.21	1.27
$V_{S,min}$	More Lewis basic (↓) O-site	-0.34	-0.36	-0.37	-0.37	-0.38
$\bar{I}_{S,min}$	More Lewis basic (↓) O-site	7.57	7.47	7.36	7.31	7.38

Supplementary references

1. Trotochaud, L., Young, S. L., Ranney, J. K. & Boettcher, S. W. Nickel-Iron Oxyhydroxide Oxygen-Evolution Electrocatalysts: The Role of Intentional and Incidental Iron Incorporation. *J. Am. Chem. Soc.* **136**, 6744–6753 (2014).
2. Rehr, J. J., Kas, J. J., Vila, F. D., Prange, M. P. & Jorissen, K. Parameter-free calculations of X-ray spectra with FEFF9. *Phys. Chem. Chem. Phys.* **12**, 5503–5513 (2010).
3. Sjoberg, P., Murray, J. S., Brinck, T. & Politzer, P. Average local ionization energies on the molecular surfaces of aromatic systems as guides to chemical reactivity. *Can. J. Chem.* **68**, 1440–1443 (1990).
4. Brinck, T., Carlqvist, P. & Stenlid, J. H. Local Electron Attachment Energy and Its Use for Predicting Nucleophilic Reactions and Halogen Bonding. *J. Phys. Chem. A* **120**, 10023–10032 (2016).
5. Brinck, T. & Stenlid, J. H. The Molecular Surface Property Approach: A Guide to Chemical Interactions in Chemistry, Medicine, and Material Science. *Adv. Theory Simulations* **2**, 1800149 (2019).
6. Halldin Stenlid, J., Johansson, A. J. & Brinck, T. The local electron attachment energy and the electrostatic potential as descriptors of surface–adsorbate interactions. *Phys. Chem. Chem. Phys.* **21**, 17001–17009 (2019).
7. Tissot, H. *et al.* Interaction of Atomic Hydrogen with the Cu2O(100) and (111) Surfaces. *J. Phys. Chem. C* **123**, 22172–22180 (2019).
8. Marcus, Y. & Marcus, Y. *Ions in Solution and Their Solvation*. (John Wiley & Sons, Inc., 2015).
9. Friebel, D. *et al.* Identification of Highly Active Fe Sites in (Ni,Fe)OOH for Electrocatalytic Water Splitting. *J. Am. Chem. Soc.* **137**, 1305–1313 (2015).
10. Görlin, M. *et al.* Tracking Catalyst Redox States and Reaction Dynamics in Ni–Fe Oxyhydroxide Oxygen Evolution Reaction Electrocatalysts: The Role of Catalyst Support and Electrolyte pH. *J. Am. Chem. Soc.* **139**, 2070–2082 (2017).
11. Bediako, D. K. *et al.* Structure–Activity Correlations in a Nickel–Borate Oxygen Evolution Catalyst. *J. Am. Chem. Soc.* **134**, 6801–6809 (2012).
12. Capehart, T. W., Corrigan, D. A., Conell, R. S., Pandya, K. I. & Hoffman, R. W. In situ extended x-ray absorption fine structure spectroscopy of thin-film nickel hydroxide electrodes. *Appl. Phys. Lett.* **58**, 865–867 (1991).
13. Baes, C. F. & Mesmer, R. S. *The Hydrolysis of Cations*. *Berichte der Bunsengesellschaft für physikalische Chemie* **81**, (John Wiley & Sons, Ltd, 1976).
14. Grimme, S., Antony, J., Ehrlich, S. & Krieg, H. A consistent and accurate ab initio parametrization of density functional dispersion correction (DFT-D) for the 94 elements H-Pu. *J. Chem. Phys.* **132**, 154104 (2010).
15. Grimme, S., Ehrlich, S. & Goerigk, L. Effect of the damping function in dispersion corrected density functional theory. *J. Comput. Chem.* **32**, 1456–1465 (2011).
16. Mathew, K., Sundararaman, R., Letchworth-Weaver, K., Arias, T. A. & Hennig, R. G. Implicit solvation model for density-functional study of nanocrystal surfaces and reaction pathways. *J. Chem. Phys.* **140**, 84106 (2014).
